# Transporting randomized trial results to estimate counterfactual survival functions in target populations

**DOI:** 10.1002/pst.2354

**Published:** 2024-01-17

**Authors:** Zhiqiang Cao, Youngjoo Cho, Fan Li

**Affiliations:** 1 Department of Mathematics, College of Big Data and Internet, Shenzhen Technology University, Shenzhen, People’s Republic of China; 2 Department of Applied Statistics, Konkuk University, Seoul, Republic of Korea; 3 Department of Biostatistics, Yale University School of Public Health, New Haven, Connecticut, USA; 4 Center for Methods in Implementation and Prevention Science, Yale University School of Public Health, New Haven, Connecticut, USA

**Keywords:** inverse odds of sampling weighting, inverse probability of censoring weighting, survey sample, survival analysis, transportability

## Abstract

When the distributions of treatment effect modifiers differ between a randomized trial and an external target population, the sample average treatment effect in the trial may be substantially different from the target population average treatment, and accurate estimation of the latter requires adjusting for the differential distribution of effect modifiers. Despite the increasingly rich literature on transportability, little attention has been devoted to methods for transporting trial results to estimate counterfactual survival functions in target populations, when the primary outcome is time to event and subject to right censoring. In this article, we study inverse probability weighting and doubly robust estimators to estimate counterfactual survival functions and the target average survival treatment effect in the target population, and provide their respective approximate variance estimators. We focus on a common scenario where the target population information is observed only through a complex survey, and elucidate how the survey weights can be incorporated into each estimator we considered. Simulation studies are conducted to examine the finite-sample performances of the proposed estimators in terms of bias, efficiency and coverage, under both correct and incorrect model specifications. Finally, we apply the proposed method to assess transportability of the results in the Action to Control Cardiovascular Risk in Diabetes—Blood Pressure (ACCORD-BP) trial to all adults with Diabetes in the United States.

## 1 | INTRODUCTION

In comparative effectiveness research, randomized controlled trials (RCTs) are often considered as the gold standard for treatment comparisons since randomization effectively removes confounding. In the ideal case where participants in RCTs are representative of the target population of scientific interest, the sample average treatment effect (SATE) is considered unbiased for the target population average treatment effect (TATE).^1^ However, participants in RCTs often may not well represent the target population due to various reasons, such as convenience sampling or patient proximity to study sites, among others. For example, African–American and Hispanic women, as well as patients over 40 years were relatively underrepresented in several past clinical trials from the AIDS Clinical Trials Group, compared with larger national populations of individuals living with human immunodeficiency virus (HIV).^2^ In practice, nonrandom participation can lead to substantial differences in the baseline demographics between the trial and target population. Because of such differences, RCTs may suffer from poor external validity and therefore can often only provide unbiased estimates for the SATE.^3–5^ Specifically, if there exist treatment effect modifiers whose distributions differs between the RCTs and the target population, then the target population average treatment effect (TATE) differs from the SATE, and accurate estimation of TATE requires adjusting for the differential distribution of effect modifiers. Due to the scientific relevance of TATE, generalizing or transporting trial results to external target populations has become an active pursuit in increasingly many scientific studies, especially in the context of public health, medicine, and implementation science research.

A popular approach to generalize findings from RCTs to target populations is through the sampling score, which is defined as the probability of participation in the trial conditional on covariates explaining the trial participation process.^2^ The sampling score plays a critical role in that conditional on the same value of the sampling score, the trial and population become exchangeable. The value of sampling score is often not known and must be estimated from the data. Under a nested design where the trial is nested within or directly sampled from the target population, the inverse probability of sampling weights have been proposed for generalizability analyses.^2,4–6^ For example, Stuart et al.^5^ applied the inverse probability of sampling weights to generalize findings in a randomized experiment of educational program to a larger target population. To improve the statistical efficiency and robustness under model misspecification, Dahabreh et al.^7^ investigated doubly robust (DR) estimators for generalizability under a nested trial design. By additionally exploiting the information from direct outcome regression, the DR estimator enables consistent estimation of the population causal effect if either the sampling score or the outcome model is correctly specified, but not necessarily both. Since we do not know which model is correctly specified in practice, the DR estimator provides some degree of protection against model misspecification, and is usually more efficient than weighting alone. For nonnested designs where the trial sample and sample from the target population are obtained separately,^8^ Buchanan et al.^2^ and Li et al.^9^ developed the weighting, regression, and DR estimators, all of which accounted for the information on target population size. When the observational sample is taken based on a complex survey, Ackerman et al.^10^ further modified the weighting estimator to combine the sampling score and known complex survey weights. Lee et al.^11^ and Li et al.^12^ recently developed calibration weighting estimator for generalizability analyses, which can improve upon the usual DR estimators by enforcing the covariate balance between the RCT and target population.

In addition to generalizability, there is a closely related literature on transportability. As pointed out in Dahabreh and Hernân,^8^ a possible conceptual difference between generalizability and transportability is that the former extends results from a randomized trial to a larger population nesting the trial population—that is, from which the trial population is sampled, whereas the latter extends result from a randomized trial to an external population defined at a potentially different place or in a different time frame. In the latter case, the external population may not even be the population from which the trial participants are directly sampled from but represents the scientific interest. While there is little practical data difference between generalizability and transportability in a sense that full data for RCT and covariates on all or a subset of nonparticipants are required for conducting the analyses, their conceptual difference necessitates a slightly different formulation of the target estimand and hence often requires a different weighting strategy.^12^ Westreich et al.^13^ developed the inverse odds of sampling weights to transport trial results to an external target population, and Dahabreh et al.^14^ developed the DR estimator to improve upon the inverse odds of sampling weighting estimator. Rudolph and van der Laan^15^ discussed targeted maximum likelihood estimators for transporting the intention-to-treat average effect, the average effect due to the actual treatment, and the complier average treatment effect under a nonnested design with patient noncompliance by extending the transport formula of Pearl and Bareinboim.^16^ Their estimator is doubly or multiply robust and may require an additional model for the actual treatment received. More recently, Josey et al.^17^ discussed how entropy balancing, as a calibration weighting estimator, can be used for transporting treatment effects from a trial population into an external target population. Despite this increasingly rich literature on transportability, the majority of the development has assumed a continuous or binary primary outcome in the trial and relatively little attention has been given to transporting trial results with a right-censored time-to-event outcome to an external population.

Our motivating application is the transportability of experimental results from the Action to Control Cardiovascular Risk in Diabetes—Blood Pressure (ACCORD-BP) trial to the U.S. population of adults with diabetes, with a primary time-to-event composite outcome subject to right-censoring. In the U.S., the effectiveness of intervention for controlling blood pressure represents an important question for people with diabetes mellitus to prevent downstream cardiovascular morbidity and mortality. In the Systolic Blood Pressure Intervention (SPRINT) trial, a significant reduction of mortality was observed when using intensive treatment for high-risk individuals without diabetes to compared with standard BP control (systolic target <120 vs. <140 mm Hg). However, results from the ACCORD-BP trial based on individuals with diabetes have shown that there was no significant improvement for using intensive treatment with respect to the composite time-to-event outcome of nonfatal myocardial infarction (MI), nonfatal stroke, or death from cardiovascular causes. These different findings result in ambiguous guidelines for blood pressure treatment, likely due to different target populations studied in different RCTs. To address this issue, Berkowitz et al.^18^ used transportability methods to re-weight individual patients from the ACCORD-BP trial of intensive versus standard treatment to better represent the demographic and clinical risk factors of the U.S. population of adults with diabetes. They combined the inverse of odds of selection weights with either the Kaplan–Meier estimator to estimate the counterfactual survival functions in the target population^19^ or the marginal structural Cox proportional hazards model to estimate the causal hazard ratio in the target population.^4^ While operationally convenient, a potential limitation of these approaches is that they have assumed that the right censoring is completely independent of all other variables and have not addressed the potential covariate-dependent censoring. Furthermore, the interpretation of the hazard ratio as a causal effect measure for the target population can be ambiguous due to the built-in selection bias.^20^

A related work that addressed covariate-dependent censoring for extending randomized trial results to target populations is the calibration approach developed in Lee et al.^21^ By combining the inverse probability of sampling weights, inverse probability of treatment weighting, and inverse probability of censoring weighting, they proposed a semiparametric estimator through the efficient influence function for generalizing survival functions and restricted mean survival time from trials to target population that includes the trial population. The proposed estimator is DR since it is consistent for the target population estimands if either the survival model or the weighting model is correctly specified and is locally efficient when both are correct. The calibration weighting approach has also been generalized to address transportability to an external target population.^22^ In this article, we consider both the weighting and DR methods for transporting trial results to estimate the counterfactual survival functions in the target population. Different from the calibration weighting estimators developed in Lee et al.,^21,22^ we investigate the use of simpler estimators that are based on explicitly estimating the weights or modeling the observed survival outcome. Specifically, we combine the inverse odds of sampling weights and the inverse probability of censoring weights^23^ to simultaneously address selection bias due to trial participation and right censoring. Furthermore, we address the common scenario where the information from the target population may only be available through a complex survey. In our application, the subjects in the NHANES study can be treated as representative sample of target population, resembling the application in Berkowitz et al.^18^ and Josey et al.,^17^ after applying the complex survey weights. We then discuss how to incorporate survey weights into the weighting and DR estimators for transportability of counterfactual survival functions. Finally, we develop approximate variance estimators to address the uncertainty of the target counterfactual survival function estimators.

The remaining parts of this article are organized as follows. In Section 2, we set up the required notations and state the related assumptions. We describe the inverse probability weighting and DR methods for transportability to estimate the target counterfactual survival functions in Section 3 and derive their approximate variance estimators in Section 4. In Section 5, we report on numerical studies to examine the finite-sample performance of the proposed point and variance estimators under both correct and incorrect model specifications, and different censoring mechanisms. The application of our methods to transport the causal survival difference between treatment and control groups found in ACCORD-BP trial to the external U.S. population is presented in Section 6. Section 7 concludes with a short discussion.

## 2 | NOTATION, ESTIMAND AND ASSUMPTIONS

Suppose the scientific interest lies in transporting the counterfactual survival functions and their difference from a randomized trial to an external target population. In this article, we consider a nonnested design, where the target population is represented by nonrandomized individuals, which may be different from the individuals in the randomized trial. Define the trial participation indicator as *S* with *S* = 1 denoting the individual participants in the randomized trial and *S* = 0 otherwise. Let X be a *p*-vector of baseline covariates including effect modifiers for individuals in the trial and those in the target population. For participants in the trial, denote *A* as the binary treatment assignment, that is, A∈{0,1}, and *A* = 1 refers to active treatment and *A* = 0 usual care. We let *T* be the time to event of interest and *C* be the censoring time. As in standard survival analysis, we cannot fully observe survival time *T* for all individuals due to right censoring; instead we observe the censoring indicator Δ=I(T≤C) and observed failure time *U* = *T* ∧ *C*, where *I*(·) represents an indicator function, and ‘^’ represents the minimum of two values. We pursue the potential outcomes framework, and let $T^{\left(a\right)}(a=0,1)$ denote the potential (or counterfactual) survival time and *C*^(*a*)^ as the potential censoring time of each trial participant if, possibly contrary to fact, s/he received treatment *A* = *a*. Thus, the causal parameter of interest is the Target Average Survival Treatment Effect (TASTE), which we define as,

$$\delta (t)=P\left(T^{\left(1\right)}\geq t|S=0\right)-P\left(T^{\left(0\right)}\geq t|S=0\right),\;\;=E\left\{I\left(T^{\left(1\right)}\geq t\right)|S=0\right\}-E\left\{I\left(T^{\left(1\right)}\geq t\right)|S=0\right\},\;\;={𝒮}_{1}(t)-{𝒮}_{0}(t),$$

where ${𝒮}_{1}(t)=E\{I(T^{\left(1\right)}\geq t)|S=0\}$ and ${𝒮}_{0}(t)=E\{I(T^{\left(0\right)}\geq t)|S=0\}$ are referred to as the target counterfactual survival functions. To estimate the causal contrast δ(t), the key is to identify the counterfactual survival functions, ${𝒮}_{a}(t)$, for a∈{0,1}.

Under the Stable Unit Treatment Value Assumption (SUTVA),^24^ the observed failure time and censoring time of each trial participant are given as $T=AT^{\left(1\right)}+(1-A)T^{\left(1\right)}$ and $C=AC^{\left(1\right)}+(1-A)C^{\left(0\right)}$, respectively. However, for the external target population, we do not observe either the treatment or outcome. Assuming that the sample sizes of target population and RCTs are *N* and *n*, respectively; usually, *N* is much larger than *n* in practice so that we do not obtain **X** for all individuals in target population. Instead, a well-designed survey sample from the target population with known survey weight is available. We assume the size of the survey sample is *m* and define *D* as inclusion indicator of an individual of combined sample (the union of the RCT and the survey sample). Note that all trial participants are included in the combined sample such that *D* = 1 whenever *S* = 1, but only *m* out of *N* individuals from the external target population are included in the combined sample; that is *D* = 1 only for the *m* individuals included in the survey sample and *D* = 0 for the remaining *N* – *m* individuals in the target population. Thus, we have P(D=1∣S=1)=1 and P(D=1∣S=0)=b where b=m/N is positive number between 0 and 1. In the special case where covariates or effect modifiers are measured for all nonrandomized individuals in the external target population, we have *m = N* and *b = 1*.

In summary, we observe information on ${𝒪}_{\text{trial}}=\left\{U_{i},\Delta_{i},A_{i},X_{i},D_{i}=1,S_{i}=1:i=1,\ldots ,n\right\}$, for trial participants, and information on ${𝒪}_{\text{pop}}=\left\{X_{i},D_{i}=1,S_{i}=0:i=n+1,\ldots ,n+m\right\}$, for nonrandomized individuals possibly through a survey sample. Now the total nonrandomized individuals is *N* ≫ *m*, and we assume that a complex survey is used to generate the observed nonrandomized individuals. We denote the survey sampling probability as *p_i_* for i=n+1,…,n+m, and therefore the known survey weight is 1/*p_i_*. Moreover, survey sampling probability *p_i_* may either be uniform or depend on covariates, but is assumed to be given or estimated prior to the transportability analyses. In our setting, since we do not observe all potential outcomes in the target population, we make the following assumptions for the identification of the counterfactual survival function ${𝒮}_{a}(t)$ and the TASTE estimand δ(t):

**Assumption 1.** (Randomization). $A_{i}⊥\left\{T_{i}^{\left(0\right)},T_{i}^{\left(1\right)},C_{i}^{\left(0\right)},C_{i}^{\left(1\right)}\right\}|S_{i}=1$, and the probability of treatment assignment in the RCT is a value between 0 and 1, that is, $0<P\left(A_{i}=1|S_{i}=1\right)=\pi <1$.

**Assumption 2.** (Strongly ignorable participation). $S_{i}⊥\left\{T_{i}^{\left(0\right)},T_{i}^{\left(1\right)},C_{i}^{\left(0\right)},C_{i}^{\left(1\right)}\right\}|X_{i}$, and the sampling score is bounded between 0 and 1 such that $0<\omega \left(X_{i}\right)=P\left(S_{i}=1|X_{i}\right)<1$.

**Assumption 3.** (Covariate-dependent censoring). $C_{i}^{\left(a\right)}⊥T_{i}^{\left(a\right)}|\left\{X_{i},S_{i}=1\right\}$ for *a* = 0, 1. This condition also implies conditional independence between the observed failure and censoring times within each arm of the randomized trial, that is, $C_{i}⊥T_{i}|\left\{X_{i},S_{i}=1,A_{i}=a\right\}$.

As we are primarily interested in transporting the trial results to external target populations so that Assumption 1 holds in the RCT by design. Assumption 2 requires exchangeability in the potential outcomes between trial participants and nonparticipants conditional on the baseline covariates **X**, and this assumption also requires that there is a positive probability of participants in the trial for each value of the covariates.^25^ While the conditional exchangeability assumption is recognized as an unverifiable assumption and may merit sensitivity analysis,^26^ the positivity assumption can often be checked by visualizing the distribution of the estimated sampling scores.^5,9^ Importantly, the positivity assumption also depends critically on the definition of target population. In cases where some nonparticipants in the external target population can never be included in the trial due to stringent inclusion and exclusion criteria, the positivity assumption may be violated and one should consider refining the external target population to avoid excessive extrapolation.^27^ Assumptions 3 is the usual assumption of noninformative censoring commonly assumed in causal survival analysis.^28,29^ Together these three key identification assumptions imply that $\left\{S_{i},C_{i}^{\left(a\right)}\right\}⊥T_{i}^{\left(a\right)}|X_{i}$, which is a version of monotone coarsening at random in the nomenclature of Tsiatis.^30^ This is because (i) when *S_i_* = 0 and $D_{i}=1,T_{i}^{\left(a\right)}$ is completely missing but **X**_*i*_ is observed, which is a many-to-one function of the full data; (ii) when *S_i_* = 1 and $C_{i}^{\left(a\right)}=t<T_{i}^{\left(a\right)}$, the many-to-one function that one observes is $\left\{I\left(T_{i}^{\left(a\right)}\geq t\right),X_{i}\right\}$ for *A_i_* = *a*; (iii) when *S_i_* = 1 and $C_{i}^{\left(a\right)}>T_{i}^{\left(a\right)}$, then one observes the complete data $\left\{T_{i}^{\left(a\right)},X_{i}\right\}$ for *A_i_* = *a*. Therefore the observed data for subject *i* is the most coarsened when *S_i_* = 0 and *D_i_* = 1, less coarsened when *S_i_* = 1 but $C_{i}^{\left(a\right)}=t_{1}<T_{i}^{\left(a\right)}$ for *A_i_* = *a*, even less coarsened when *S_i_* = 1 but $C_{i}^{\left(a\right)}=t_{2}<T_{i}^{\left(a\right)}\left(t_{1}<t_{2}\right)$ for *A_i_* = *a*, and not coarsened at all when *S_i_* = 1 but $C_{i}^{\left(a\right)}>T_{i}^{\left(a\right)}$.

In the more practical scenario where covariate information for the external target population is observed only through a selected subset of size *m*, we assume that this subset is selected according to a complex survey with survey weights that are either known or estimated from prior data. In other words, we do not pursue the more complicated scenario where we have access to additional data to estimate the survey weights, but instead assume that these weights are already well-estimated and treated as given. This helps us focus on the transportability aspect of the problem and we will return to a discussion in Section 7. We formalize this assumption as follows.

**Assumption 4.** (Survey design). Among the nonrandomized participants, the information of covariates is observed through a subset that is selected according to given survey weights, $P\left(D_{i}=1|S_{i}=0,X_{i}\right)=p\left(X_{i}\right)=p_{i}$.

Based on Assumption 4, the design-weighted observational sample can be representative of the target population. In the special case where $P\left(D_{i}=1|S_{i}=0,X_{i}\right)=p$ with *p* as a constant defined as *m/N*, the survey design reduces to a simple random sampling design that does not depend on any covariates. Buchanan et al.^2^ and Li et al.^9^ considered such a simple random sampling design for generalizability analyses with noncensored outcomes.

## 3 | ESTIMATING TARGET COUNTERFACTUAL SURVIVAL FUNCTIONS AND TARGET AVERAGE SURVIVAL TREATMENT EFFECTS

### 3.1 | Preliminaries

To begin with, we consider the scenario where covariate information among the external target population is fully observed. In this case, we have *m* = *N* since no survey sampling is considered among the target population, Assumptions 1–3 are sufficient to identify the target counterfactual survival function. Extensions to estimators in case where nonrandomized individuals come from a complex survey will be discussed in Section 3.4.

We consider weighting estimators and DR estimators of δ(t) to transport trial results to a target population. The weighting approach first requires modeling the sampling score to derive the inverse odds of sampling weights. We assume the sampling score $\omega \left(X_{i}\right)$ follows a logistic regression with a finite-dimensional parameter vector, that is,

$$\omega \left(X_{i}\right)=P\left(S_{i}=1|X_{i}\right)=\frac{exp\left(\theta_{0}+X_{i}^{T}\theta_{1}\right)}{1+exp\left(\theta_{0}+X_{i}^{T}\theta_{1}\right)},$$

where $\theta ={\left(\theta_{0},\theta_{1}^{T}\right)}^{T}$ is a vector of regression coefficients to be estimated, **X** is a vector made up of (possibly transformed) baseline covariates or effect modifiers. Let $\hat{\theta }$ denote the maximum likelihood estimator of ***θ***. For each observation in the trial and nonrandomized sample, we denote the estimated sampling score as $\hat{\omega }\left(X_{i}\right)$.

In addition, the weighting approach requires accounting for covariate-dependent censoring through the inverse probability of censoring weights.^23^ To do so, we use Cox proportional hazards model^31^ to model distribution of censoring time *C_i_* given treatment *A_i_* = *a* and covariates in the trial population, that is,

(1) $$\lambda_{a}^{C}\left(t|X_{i},S_{i}=1\right)=\lambda_{0a}^{C}(t)exp\left(X_{i}^{T}\beta_{a}\right),$$

where $\lambda_{0a}^{C}(t)$ is an unspecified treatment-specific baseline hazard function, and $\beta_{a}$ is an unknown regression coefficient vector for each treatment group. The estimator ${\hat{\beta }}_{a}$ can be obtained by maximizing the corresponding partial likelihood function.^32^ Then, we can use the Breslow estimator to estimate the cumulative hazard function $\Lambda_{0a}^{C}(t)=\int_{0}^{t}\lambda_{0a}^{C}(u)du$, denoted as ${\hat{\Lambda }}_{0a}^{C}(t)$, and obtain the individual-specific cumulative hazard function as ${\hat{\Lambda }}_{a}^{C}\left(t|X_{i},S_{i}=1\right)={\hat{\Lambda }}_{0a}^{C}(t)exp\left(X_{i}^{T}{\hat{\beta }}_{a}\right)$. Let $K_{a}\left(t|X_{i}\right)=P\left(C_{i}^{\left(a\right)}\geq t|X_{i},S_{i}=1\right)=P\left(C_{i}\geq t|X_{i},A_{i}=a,S_{i}=1\right)$, then the estimated survival function of the censoring time for individual i at t is ${\hat{K}}_{a}\left(t|X_{i}\right)=exp\left\{-{\hat{\Lambda }}_{a}^{C}\left(t|X_{i},S_{i}=1\right)\right\}$, which forms the basis of constructing the inverse probability of censoring weights.

Beyond the weighting approach, which requires modeling the sampling score and the censoring distribution, the direct outcome modeling approach involves modeling the observed failure time as a function of covariates. Specifically, for distribution of survival time, we can also use a similar Cox model (1) to obtain the corresponding conditional survival function. Denote $H_{a}\left(t|X_{i}\right)=P\left(T_{i}^{\left(a\right)}\geq t|X_{i}\right)=P\left(T_{i}^{\left(a\right)}\geq t|X_{i},S_{i}=1\right)=P\left(T_{i}\geq t|X_{i},A_{i}=a,S_{i}=1\right)$, after obtaining cumulative hazard function of $T_{i}^{\left(a\right)}$, that is, ${\hat{\Lambda }}_{a}\left(t|X_{i}\right)$, then ${\hat{H}}_{a}\left(t|X_{i}\right)=exp\left\{-{\hat{\Lambda }}_{a}\left(t|X_{i}\right)\right\}$. Alternative to the Cox model, one can also use an accelerated failure time (AFT) model^33^ to estimate the survival function of survival time *T_i_* or censoring time *C_i_*. For example, the form of AFT model for $T_{i}^{\left(a\right)}$ can be written as:

$$log\left(T_{i}^{\left(a\right)}\right)=\gamma_{0a}+X_{i}^{T}\gamma_{1a}+\sigma_{a}\epsilon_{i}^{\left(a\right)},$$

where $\gamma_{a}={\left(\gamma_{0a},\gamma_{1a}^{T}\right)}^{T}$ is the vector of regression parameters under treatment $A=a,\epsilon_{i}^{\left(a\right)}$ follows parametric distribution, such as standard extreme value (Weibull regression), standard normal (log-normal regression) or standard logistic (log-logistic regression) distribution that determines the shape of the survival function for treatment group *A* = *a*, and $\sigma_{a}$ is a scale parameter which determines variability of survival distributions from $\epsilon_{ia}$. We can estimate the survival function of the failure time as follows:

$${\hat{H}}_{a}\left(t|X_{i}\right)=P\left(\epsilon_{i}^{\left(a\right)}\geq \frac{logt-{\hat{\gamma }}_{0a}-X_{i}^{T}{\hat{\gamma }}_{1a}}{{\hat{\sigma }}_{a}}\right),$$

where ${\left({\hat{\gamma }}_{0a},{\hat{\gamma }}_{1a},{\hat{\sigma }}_{a}\right)}^{T}$ are estimated parameters by standard maximum likelihood method by using ${𝒪}_{\text{trial}}$. We can compute the survival probability easily by using the specified distribution. In the above sampling score model, censoring time model, and failure outcome model, we use the same set of covariates (i.e., **X**) throughout for simplicity. However, it is possible in practice that a different subset of **X** contributes to each model that describes a specific aspect of the data generating process. In principle, one should include all effect modifiers and covariates that affect trial participation into the sampling score model, include all effect modifiers and prognostic covariates into the failure outcome model, and include all variables that are predictive of censoring and survival into the censoring time model. The selection of these variables may require discussions with the content matter experts and can be assisted by data-driven variable selection techniques. We return to a discussion of potential variable selection methods in Section 7. Finally, when certain covariates are highly skewed, Box–Cox transformation for **X** may be considered to improve the stability and fit of the sampling score model, censoring time model, and failure outcome model. With these preliminary discussions on possible modeling steps, we next introduce the proposed transportability estimators.

### 3.2 | Inverse odds of sampling weighting and inverse probability of censoring weighting estimators

To estimate the target counterfactual survival functions, we first consider weighting estimators that combine the inverse odds of sampling weights and inverse probability of censoring weights, which we refer to the inverse probability weighting (IPW) estimators in this article hereafter. The first IPW estimator, denoted as IPW1 estimator, is akin to the Horvitz–Thompson estimator is given as follows:

(2) $${\hat{𝒮}}_{a}^{\text{IPW}1}(t)=\frac{1}{m}\overset{n+m}{\underset{i=1}{\sum }}\frac{S_{i}I\left(A_{i}=a\right)\left\{1-\hat{\omega }\left(X_{i}\right)\right\}{\text{Δ}}_{i}I\left(U_{i}\geq t\right)}{\pi_{a}\hat{\omega }\left(X_{i}\right){\hat{K}}_{a}\left(U_{i}|X_{i}\right)},$$

where $\pi_{a}=\pi I(a=1)+(1-\pi )I(a=0)$ is the (known) randomization proportion to arm a∈{0,1} in the RCT. The proof of consistency for IPW1 estimator is provided in the Web Appendix A.1. This is a simple estimator where the weight for each observation is not normalized such that ${\hat{𝒮}}_{a}^{IPW1}(t)$ may have large variability when the estimated censoring survival function ${\hat{K}}_{a}\left(U_{i}|X\right)$ or the sampling score $\hat{\omega }(X)$ approaches zero. As a potential improvement and observing the following identity:

$$E\left\{\frac{S_{i}I\left(A_{i}=a\right)}{\pi_{a}\omega \left(X_{i}\right)}\right\}=1,$$

we can construct a bounded inverse odds of sampling weights that is akin to the Hajek estimator in the survey sampling literature, defined as IPW2, that is,

(3) $${\hat{𝒮}}_{a}^{IPW2}(t)={\left\{\sum_{i=1}^{n+m}\frac{S_{i}I\left(A_{i}=a\right)\left\{1-\hat{\omega }\left(X_{i}\right)\right\}}{\hat{\omega }\left(X_{i}\right)}\right\}}^{-1}\sum_{i=1}^{n+m}\frac{S_{i}I\left(A_{i}=a\right)\left\{1-\hat{\omega }\left(X_{i}\right)\right\}\Delta_{i}I\left(U_{i}\geq t\right)}{\hat{\omega }\left(X_{i}\right){\hat{K}}_{a}\left(U_{i}|X_{i}\right)}.$$

By normalizing the inverse odds of sampling weights with respect to the sampling score $\omega \left(X_{i}\right)$, the IPW2 estimator has an opportunity to improve the stablility of transportability inference when the sampling score is estimated to be close to 0. Of note, the randomization probability *π_a_* vanished in the IPW2 estimator due to weight normalization. The proof of consistency for IPW2 estimator is similar to that of IPW1, and is thus omitted for brevity.

Notice that the set of IPW estimators only requires modeling the sampling score and the censoring survival functions, but not the failure time outcome distribution. When both models for $K_{a}\left(U_{i}|X_{i}\right)$ and $\omega \left(X_{i}\right)$ are correctly specified, ${\hat{𝒮}}_{a}^{\text{IPW1}}(t)$ and ${\hat{𝒮}}_{a}^{\text{IPW2}}(t)$ are both pointwise consistent to the target counterfactual survival function ${𝒮}_{a}(t)$ across $t\in \left[0,\tau \right]$ with τ being the maximum follow-up time. The difference in the estimated target counterfactual survival function then provides a consistent estimator for TASTE *δ*(*t*).

Finally, the adequacy of the fitted sampling score model may be checked via weighted covariate balance between the RCT and the external target population. Specifically for each covariate $X_{ik}(k=1,\ldots ,p)$, we can consider a similar approach to define the standardized mean difference (SMD) as follows,

(4) $$\text{SMD}=\frac{1}{S}\left|\frac{\sum_{i=1}^{n+m}S_{i}X_{ik}\tau_{i}}{\sum_{i=1}^{n+m}S_{i}\tau_{i}}-\frac{\sum_{i=1}^{n+m}\left(1-S_{i}\right)X_{ik}}{m}\right|,$$

where the denominator *s* is the standard deviation of *X_ik_* in the target population. When the weight for each RCT observation $\tau_{i}=1$, SMD quantifies the preexisting differences between the trial and external population. When $\tau_{i}=\left\{1-\hat{\omega }\left(X_{i}\right)\right\}/\hat{\omega }\left(X_{i}\right)$, the SMD measures the similarity between the inverse odds of sampling weighted trial and external population, and can be used as a diagnostic check of the sampling score weights. The usual iterative fitting-checking procedure recommended in the propensity score literature may be considered in fitting the sampling score model.^9^ That is, if the inverse odds of sampling weights ensure that the SMD for the majority of covariates falls within 20% (or 10% as a more stringent threshold), then the sampling score model may be considered adequate. In practice, one may consider including additional interaction or high-order terms to improve the weighted covariate balance if main effects logistic sampling score model does not lead to satisfactory balance.

### 3.3 | DR estimators

As an alternative to the IPW estimator, it is possible to estimate the target counterfactual survival functions through direct outcome regression. Similar to Dahabreh et al.,^14^ this simple outcome regression estimator is given as follows:

$${\hat{S}}_{a}^{OM}(t)=\frac{\sum_{i=1}^{n+m}\left(1-S_{i}\right){\hat{H}}_{a}\left(t|X_{i}\right)}{\sum_{i=1}^{n+m}\left(1-S_{i}\right)},$$

where ${\hat{H}}_{a}\left(t|X_{i}\right)$ is a regression estimator of the survival function $P\left(T_{i}\geq t|X_{i},A_{i}=a\right)$), and when $S_{i}=0,{\hat{H}}_{a}\left(t|X_{i}\right)$ is the corresponding predictions for units in the target population based on parameter estimation obtained from trial participants. As we mentioned in Section 3.1, one can use the Cox model or AFT model to estimate $P\left(T_{i}\geq t|X_{i},S_{i}=1,A_{i}=a\right)$. In this article, we are interested in leveraging the information from such an outcome model to improve upon the IPW estimators in Section 3.2. Specifically, to enhance the robustness of transportability estimators against model misspecification, according to the semiparametric efficiency theory,^30^ we can construct a DR estimator to estimate the marginal TASTE estimand, δ(t), by combining the strengths of inverse probability weighting and direct failure time outcome regression. Recall that our outcome model is $H_{a}\left(t|X_{i}\right)=P\left(T_{i}\geq t|X_{i},A_{i}=a\right)=P\left(T_{i}^{\left(a\right)}\geq t|X_{i}\right)$ and ${\hat{H}}_{a}\left(t|X_{i}\right)$ is the corresponding estimator of $\left(T_{i}^{\left(a\right)}\geq t|X_{i}\right)$. We extend the approach of Bai et al.^34^ developed for observational comparisons to transportability analyses under inverse odds of sampling weights, and find that the optimal estimating function that can lead to an augmented inverse probability weighted (AIPW) estimator is given as follows:

$$\frac{S_{i}I\left(A_{i}=a\right)\left\{1-\omega \left(X_{i}\right)I\left(U_{i}\geq t\right)\right\}}{\pi_{a}\omega \left(X_{i}\right)K_{a}\left(t|X_{i}\right)}-\left(\frac{S_{i}I\left(A_{i}=a\right)\left(1-\omega \left(X_{i}\right)\right)}{\omega \left(X_{i}\right)\pi_{a}}\right)H_{a}\left(t|X_{i}\right)\;+\frac{S_{i}I\left(A_{i}=a\right)\left\{1-\omega \left(X_{i}\right)\right\}}{\pi_{a}\omega \left(X_{i}\right)}\int_{0}^{t}\frac{H_{a}\left(t|X_{i}\right)}{H_{a}\left(u|X_{i}\right)}\frac{dM_{a}\left(u|X_{i}\right)}{K_{a}\left(u|X_{i}\right)}+\left(1-S_{i}\right)H_{a}\left(t|X_{i}\right)-{𝒮}_{a}(t),$$

where $U_{i}^{\left(a\right)}=T_{i}^{\left(a\right)}\wedge C_{i}^{\left(a\right)}$ and $\Delta_{i}^{\left(a\right)}=I\left(T_{i}^{\left(a\right)}\leq C_{i}^{\left(a\right)}\right)$, and $dM_{a}\left(u|X_{i}\right)=dN_{a,i}(u)-I\left(U_{i}^{\left(a\right)}\geq u\right)\lambda_{a}\left(u|X_{i}\right)$ is the martingale increment for the potential censoring distribution, $N_{a,i}(u)=I\left(U_{i}^{\left(a\right)}\leq u,\Delta_{i}^{\left(a\right)}=0\right)$ is the counting process for the censoring time and $\lambda_{a}\left(u|X_{i}\right)=-d\;log\;K_{a}\left(u|X_{i}\right)/du$ is hazard function for censoring distribution.

Then based on the observed data, the AIPW estimating function is given as follows:

(5) $$\frac{1}{m}\overset{n+m}{\underset{i=1}{\sum }}[\frac{S_{i}I\left(A_{i}=a\right)\left\{1-\omega \left(X_{i}\right)\right\}\left\{I\left(U_{i}\geq t\right)-K_{a}\left(t|X_{i}\right)H_{a}\left(t|X_{i}\right)\right\}}{\pi_{a}\omega \left(X_{i}\right)K_{a}\left(t|X_{i}\right)}+\frac{S_{i}I\left(A_{i}=a\right)\left\{1-\omega \left(X_{i}\right)\right\}}{\pi_{a}\omega \left(X_{i}\right)}\;\;\int_{0}^{t}\frac{H_{a}\left(t|X_{i}\right)}{H_{a}\left(u|X_{i}\right)}\frac{dM_{a}\left(u|X_{i}\right)}{K_{a}\left(u|X_{i}\right)}+\left(1-S_{i}\right)H_{a}\left(t|X_{i}\right)]-{𝒮}_{a}(t)=0.$$

If the sampling score $\omega \left(X_{i}\right)$, the conditional censoring distribution $K_{a}\left(t|X_{i}\right)$ and the conditional survival distribution $H_{a}\left(t|X_{i}\right)$ are estimated from the data, then we can use Equation (5) to obtain the AIPW estimator as follows:

(6) $${\hat{𝒮}}_{a}^{\text{DR}1}(t)=\frac{1}{m}\overset{n+m}{\underset{i=1}{\sum }}\left[\frac{S_{i}I\left(A_{i}=a\right)\left\{1-\hat{\omega }\left(X_{i}\right)\right\}\left\{I\left(U_{i}\geq t\right)-{\hat{K}}_{a}\left(t|X_{i}\right){\hat{H}}_{a}\left(t|X_{i}\right)\right\}}{\pi_{a}\hat{\omega }\left(X_{i}\right){\hat{K}}_{a}\left(t|X_{i}\right)}+\frac{S_{i}I\left(A_{i}=a\right)\left\{1-\hat{\omega }\left(X_{i}\right)\right\}}{\pi_{a}\hat{\omega }\left(X_{i}\right)}\int_{0}^{t}\frac{d{\hat{M}}_{a,i}\left(u|X_{i}\right)}{{\hat{K}}_{a}\left(u|X_{i}\right)}\frac{{\hat{H}}_{a}\left(t|X_{i}\right)}{{\hat{H}}_{a}\left(u|X_{i}\right)}\right]\;\;+\frac{1}{m}\overset{n+m}{\underset{i=1}{\sum }}\left(1-S_{i}\right){\hat{H}}_{a}\left(t|X_{i}\right),$$

where $d{\hat{M}}_{a}\left(u|X_{i}\right)=dN_{a,i}(u)-I\left(U_{i}^{\left(a\right)}\geq u\right){\hat{\lambda }}_{a}\left(u|X_{i}\right)$ is the martingale increment with the estimated hazard function. We show in Web Appendix A.2 that this estimator is DR, that is, ${\hat{𝒮}}_{a}^{DR1}(t)$ is pointwise consistent to the target counterfactual survival function when either models for *C_i_* and $\omega \left(X_{i}\right)$ are correctly specified or the model for survival time *T_i_* given **X**_*i*_ is correctly specified. If all three working models are correct, then the resulting estimator is semiparametric efficient in that the asymptotic variance of the AIPW estimator is the smallest among a class of regular and asymptotically linear estimators.^30^

To potentially improve the above DR estimator when the estimated inverse odds of sampling weights are relatively large, we consider a normalized version of the DR estimator, in the spirit of IPW2. We term this as the DR2 estimator, which takes the below form:

(7) $${\hat{𝒮}}_{a}^{\text{DR}2}(t)=\overset{n+m}{\underset{i=1}{\sum }}\frac{S_{i}I\left(A_{i}=a\right)\left\{1-\hat{\omega }\left(X_{i}\right)\right\}\left\{I\left(U_{i}\geq t\right)-{\hat{K}}_{a}\left(t|X_{i}\right){\hat{H}}_{a}\left(t|X_{i}\right)\right\}/\left(\hat{\omega }\left(X_{i}\right){\hat{K}}_{a}\left(t|X_{i}\right)\right)}{\sum_{i=1}^{n+m}S_{i}I\left(A_{i}=a\right)\left\{1-\hat{\omega }\left(X_{i}\right)\right\}/\hat{\omega }\left(X_{i}\right)}\;\;+\overset{n+m}{\underset{i=1}{\sum }}\frac{S_{i}I\left(A_{i}=a\right)\left\{1-\hat{\omega }\left(X_{i}\right)\right\}/\hat{\omega }\left(X_{i}\right)}{\sum_{i=1}^{n+m}S_{i}I\left(A_{i}=a\right)\left\{1-\hat{\omega }\left(X_{i}\right)\right\}/\hat{\omega }\left(X_{i}\right)}\overset{t}{\underset{0}{\int }}\frac{d{\hat{M}}_{a}\left(u|X_{i}\right)}{{\hat{K}}_{a}\left(u|X_{i}\right)}\frac{{\hat{H}}_{a}\left(t|X_{i}\right)}{{\hat{H}}_{a}\left(u|X_{i}\right)}+\frac{1}{m}\overset{n+m}{\underset{i=1}{\sum }}\left(1-S_{i}\right){\hat{H}}_{a}\left(t|X_{i}\right).$$

Notice that by normalizing the weight in each arm, the DR2 estimator also does not depend on the randomization proportion $\pi_{a}$ in the RCT. As the weights are normalized, the DR2 estimator may be potentially more stable in finite samples compared with the DR1 estimator.

### 3.4 | Incorporating survey weights when information among target population is observed in a subset

In our motivating application, the external target population is only represented through a complex survey. In this Section, we will elaborate on what modifications are needed to provide valid transportability estimates using IPW and DR estimators. As we discussed in Section 2, with complex survey, our data include covariate information from survey sample, whose sample size is *m* and *N* is much larger than *m*. For identification of our target estimates, specifically, we will assume Assumption 4 and assume that the survey weight (1/*p_i_*) is given for each participant in the nonrandomized cohort survey sample.

Once the sampling score is estimated from the observed data, we further observe that

$$E\left\{\left(1-S_{i}\right)p_{i}^{-1}\right\}=P\left(S_{i}=0\right).$$

Based on the survey sample, estimation of ω(X) should be based on the weighted logistic regression with individual weight 1/*p_i_* for the nonrandomized sample and weight 1 for the RCT sample^35^ because the observed survey sample itself is not representative of the target population. Specifically, we adopt the weighted logistic regression similar to Li et al.^9^ and Ackerman et al.^10^ and predict the sampling score for each observation. Then, the IPW1 estimator should further incorporate the survey weights through

$${\hat{𝒮}}_{a}^{\text{IPW}1}(t)=\frac{1}{\overset{n+m}{\underset{i=1}{\sum }}\left(1-S_{i}\right)p_{i}^{-1}}\overset{n+m}{\underset{i=1}{\sum }}\frac{S_{i}I\left(A_{i}=a\right)\left\{1-\hat{\omega }\left(X_{i}\right)\right\}{\text{Δ}}_{i}I\left(U_{i}\geq t\right)}{\pi_{a}\hat{\omega }\left(X_{i}\right){\hat{K}}_{a}\left(U_{i}|X_{i}\right)}.$$

Interestingly, as long as the sampling score estimation accounts for the survey weights through the weighted logistic regression, IPW2 estimator remains identical to Equation (3) in its construction. In other words, the survey weights are applied twice for IPW1 estimator (in both the weighted logistic regression for estimating sampling scores and the construction of the final estimator), whereas the survey weights are applied only once for the IPW2 estimator (only in the weighted logistic regression for estimating sampling scores). Since the survey weights are assumed to be given (or estimated in prior studies) and the probability of treatment assignment is known in the RCT, these two IPW estimators are also consistent to the target counterfactual survival functions as long as the sampling score model and the censoring model are both correctly specified.

Of note, in the case where the target population is only observed through a survey sample, the SMD criterion to check the sampling score model fit in Equation (4) will be modified as follows:

$$\text{SMD}=\frac{1}{S}\left|\frac{\sum_{i=1}^{n+m}S_{i}X_{ik}\tau }{\sum_{i=1}^{n+m}S_{i}\tau_{i}}-\frac{\sum_{i=1}^{n+m}\left(1-S_{i}\right)X_{ik}p_{i}^{-1}}{\sum_{i=1}^{n+m}\left(1-S_{i}\right)p_{i}^{-1}}\right|.$$

Slightly different from Equation (4), the denominator s is the standard deviation of *X_ik_* in the target population estimated by,

$$S={\left\{\frac{\sum_{i=1}^{n+m}p_{i}^{-1}{\left(X_{ik}-{\bar{X}}_{k}\right)}^{2}}{\sum_{i=1}^{n+m}\left(1-S_{i}\right)p_{i}^{-1}-\frac{\sum_{i=1}^{n+m}\left(1-S_{i}\right)p_{i}^{-2}}{\sum_{i=1}^{n+m}\left(1-S_{i}\right)p_{i}^{-1}}}\right\}}^{\frac{1}{2}},$$

where ${\bar{X}}_{k}=\sum_{i=1}^{n+m}\left(1-S_{i}\right)X_{ik}p_{i}^{-1}/\sum_{i=1}^{n+m}\left(1-S_{i}\right)p_{i}^{-1}$ is the target population average. This expression is essentially the weighted standard deviation of each covariate with survey weight 1=*p_i_*.

However, IPW1 and IPW2 estimators may still be inefficient when censoring rate is large. Hence we adapt the DR estimators to accommodate complex survey data. We can additionally include the survey weights in the two DR estimators as follows:

(8) $${\hat{𝒮}}_{a}^{\text{DR}1}(t)=\frac{1}{\overset{n+m}{\underset{i=1}{\sum }}\left(1-S_{i}\right)p_{i}^{-1}}\overset{n+m}{\underset{i=1}{\sum }}[\frac{S_{i}I\left(A_{i}=a\right)\left\{1-\hat{\omega }\left(X_{i}\right)\right\}\left\{I\left(U_{i}\geq t\right)-{\hat{K}}_{a}\left(t|X_{i}\right){\hat{H}}_{a}\left(t|X_{i}\right)\right\}}{\pi_{a}\hat{\omega }\left(X_{i}\right){\hat{K}}_{a}\left(t|X_{i}\right)}+\frac{S_{i}I\left(A_{i}=a\right)\left\{1-\hat{\omega }\left(X_{i}\right)\right\}}{\pi_{a}\hat{\omega }\left(X_{i}\right)}\;\;\overset{t}{\underset{0}{\int }}\frac{d{\hat{M}}_{a}\left(u|X_{i}\right)}{{\hat{K}}_{a}\left(u|X_{i}\right)}\frac{{\hat{H}}_{a}\left(t|X_{i}\right)}{{\hat{H}}_{a}\left(u|X_{i}\right)}+\left(1-S_{i}\right)p_{i}^{-1}{\hat{H}}_{a}\left(t|X_{i}\right)],$$

and

(9) $${\hat{𝒮}}_{a}^{\text{DR}2}(t)=\overset{n+m}{\underset{i=1}{\sum }}\frac{S_{i}I\left(A_{i}=a\right)\left\{1-\hat{\omega }\left(X_{i}\right)\right\}\left\{I\left(U_{i}\geq t\right)-{\hat{K}}_{a}\left(t|X_{i}\right){\hat{H}}_{a}\left(t|X_{i}\right)\right\}/\left(\hat{\omega }\left(X_{i}\right){\hat{K}}_{a}\left(t|X_{i}\right)\right)}{\overset{n+m}{\underset{i=1}{\sum }}S_{i}I\left(A_{i}=a\right)\left\{1-\hat{\omega }\left(X_{i}\right)\right\}/\hat{\omega }\left(X_{i}\right)}\;\;+\overset{n+m}{\underset{i=1}{\sum }}\frac{S_{i}I\left(A_{i}=a\right)\left\{1-\hat{\omega }\left(X_{i}\right)\right\}/\hat{\omega }\left(X_{i}\right)}{\overset{n+m}{\underset{i=1}{\sum }}S_{i}I\left(A_{i}=a\right)\left\{1-\hat{\omega }\left(X_{i}\right)\right\}/\hat{\omega }\left(X_{i}\right)}\overset{t}{\underset{0}{\int }}\frac{d{\hat{M}}_{a}\left(u|X_{i}\right)}{{\hat{K}}_{a}\left(u|X_{i}\right)}\frac{{\hat{H}}_{a}\left(t|X_{i}\right)}{{\hat{H}}_{a}\left(u|X_{i}\right)}+\frac{\overset{n+m}{\underset{i=1}{\sum }}\left(1-S_{i}\right)p_{i}^{-1}{\hat{H}}_{a}\left(t|X_{i}\right)}{\overset{n+m}{\underset{i=1}{\sum }}\left(1-S_{i}\right)p_{i}^{-1}}.$$

Compared with the estimators Equations (6) and (7), the above two DR estimators additionally incorporate the survey weights when utilizing information from the direct outcome regression $H_{a}\left(t|X_{i}\right)$, as in the last term of Equations (8) and (9). In other words, the DR1 estimator uses the survey weights three times whereas the DR2 estimator uses the survey weights twice (also see Table 1 for a quick reference on when survey weights are used in each of the four estimators we considered). Following arguments in Web Appendix A.2, we can also show that these DR estimators are consistent to the target counterfactual survival function when the sampling score model and censoring model are correctly specified, or when the direct outcome regression model is correctly specified, but not necessarily both.

## 4 | APPROXIMATE VARIANCE ESTIMATION FOR TRANSPORTABILITY ANALYSES

We discuss strategies to approximate the variability of the IPW and DR estimator for transporting the trial evidence to estimate the target counterfactual survival functions and the TASTE estimand. In general, one can express each transportability estimator as a solution to the corresponding unbiased estimating equations to establish the asymptotic normality and hence obtain the large-sample variance. In simpler settings with noncensored outcomes, the unbiased estimating equations representation permits the derivation of a sandwich variance estimator that fully accounts for the uncertainty in estimating the parametric nuisance models.^2,9,36,37^ In this article, we focus on evaluating a simpler strategy that ignores the uncertainty in estimating the parametric sampling score model, censoring model as well as the outcome model, and instead focus on addressing the key component of the estimating function that drives the uncertainty of the transportability estimators. For IPW, it is known that such a strategy is often conservative and therefore still provides valid variance estimation. For DR estimators, this strategy is often sufficient as we also show in the ensuing simulation studies. In what follows, we mainly present approximate variance estimators for the IPW and DR estimators when the interest lies in the TASTE estimand. Approximate variance estimation for the target counterfactual survival functions follows the exact same reasoning and will be omitted for brevity. To inform our application, we also present the case with survey weights, and the case when the target population is fully observed can be considered as a special case where $p_{i}=1$ for i=n+1,…,n+m.

The main idea of variance estimation is to obtain the appropriate influence function. For the IPW1 estimator, we can rewrite the equation such that

$${\hat{𝒮}}_{a}^{\text{IPW}1}(t)-{𝒮}_{a}(t)=\frac{1}{\overset{n+m}{\underset{i=1}{\sum }}\left(1-S_{i}\right)p_{i}^{-1}}\overset{n+m}{\underset{i=1}{\sum }}\left[\frac{S_{i}I\left(A_{i}=a\right)\left\{1-\hat{\omega }\left(X_{i}\right)\right\}\Delta_{i}I\left(U_{i}\geq t\right)}{\pi_{a}\hat{\omega }\left(X_{i}\right){\hat{K}}_{a}\left(U_{i}|X_{i}\right)}-\left(1-S_{i}\right)p_{i}^{-1}{𝒮}_{a}(t)\right].$$

Therefore, according to the Central Limit Theorem, an approximate variance estimator for the IPW1 estimator for the TASTE at time $t,{\hat{\delta }}^{\text{IPW}1}(t)={\hat{𝒮}}_{1}^{\text{IPW}1}(t)-{\hat{𝒮}}_{0}^{\text{IPW}1}(t)$, can be formulated as follows:

(10) $$\text{Var}\left\{{\hat{\delta }}^{\text{IPW}1}(t)\right\}\approx \frac{1}{{\left\{\overset{n+m}{\underset{i=1}{\sum }}\left(1-S_{i}\right)p_{i}^{-1}\right\}}^{2}}\overset{n+m}{\underset{i=1}{\sum }}{\left\{{\hat{I}}_{i,1}^{\text{IPW}1}(t)-{\hat{I}}_{i,0}^{\text{IPW}1}(t)-{\hat{\delta }}^{\text{IPW}1}(t)\right\}}^{2},$$

where the approximate influence function (that ignores the variability for estimating the sampling score and censoring probability and scaled by a normalization constant) is given by as follows:

$${\hat{I}}_{i,a}^{\text{IPW}1}(t)=\frac{S_{i}I\left(A_{i}=a\right)\left\{1-\hat{\omega }\left(X_{i}\right)\right\}{\text{Δ}}_{i}I\left(U_{i}\geq t\right)}{\pi_{a}\hat{\omega }\left(X_{i}\right){\hat{K}}_{a}\left(U_{i}|X_{i}\right)}-\left(1-S_{i}\right)p_{i}^{-1}{\hat{𝒮}}_{a}^{\text{IPW}1}(t).$$

Using a similar approach, the approximate variance estimator for the IPW2 transportability estimator ${\hat{\delta }}^{\text{IPW}2}(t)={\hat{𝒮}}_{1}^{\text{IPW2}}(t)-{\hat{𝒮}}_{0}^{\text{IPW2}}(t)$ is given as follows:

(11) $$\text{Var}\left\{{\hat{\delta }}^{\text{IPW}2}(t)\right\}\approx \overset{n+m}{\underset{i=1}{\sum }}{\left\{{\hat{I}}_{i,1}^{\text{IPW}2}(t)\right\}}^{2}+\overset{n+m}{\underset{i=1}{\sum }}{\left\{{\hat{I}}_{i,0}^{\text{IPW}2}(t)\right\}}^{2},$$

where the approximate influence function is given as follows:

$${\hat{I}}_{i,a}^{\text{IPW}2}(t)={\left[\overset{n+m}{\underset{i=1}{\sum }}\frac{S_{i}I\left(A_{i}=a\right)\left\{1-\hat{\omega }\left(X_{i}\right)\right\}}{\hat{\omega }\left(X_{i}\right)}\right]}^{-1}\times \left[\frac{S_{i}I\left(A_{i}=a\right)\left\{1-\hat{\omega }\left(X_{i}\right)\right\}\left\{{\text{Δ}}_{i}I\left(U_{i}\geq t\right)/{\hat{K}}_{a}\left(U_{i}|X_{i}\right)-{\hat{𝒮}}_{a}^{IPW2}(t)\right\}}{\hat{\omega }\left(X_{i}\right)}\right].$$

In this case, we ignore variability of censoring and sampling score. To obtain correct variance, it might be necessary to reflect uncertainty of estimation of censoring and sampling score. However, we avoid doing so in this estimation because derivation of influence function is extremely tedious with these quantities and the amount of improvement in accuracy may be minimal given sufficient sample sizes. It has been shown that ignoring variability of censoring does not have considerable effect on the coverage in two-arm observational studies.^34^ For the DR estimators, the approximate variance estimators have a slightly more complicated form due to the construction of the estimators, and we elaborate on their forms below and will examine their finite-sample performance in the ensuing simulation study. Specifically, for the DR1 estimator, we can similar rewrite the estimator as

$${\hat{𝒮}}_{a}^{\text{DR}1}(t)-{𝒮}_{a}(t)=\frac{1}{\overset{n+m}{\underset{i=1}{\sum }}\left(1-S_{i}\right)p_{i}^{-1}}\overset{n+m}{\underset{i=1}{\sum }}[\frac{S_{i}I\left(A_{i}=a\right)\left\{1-\hat{\omega }\left(X_{i}\right)\right\}\left\{I\left(U_{i}\geq t\right)-{\hat{K}}_{a}\left(t|X_{i}\right){\hat{H}}_{a}\left(t|X_{i}\right)\right\}}{\pi_{a}\hat{\omega }\left(X_{i}\right){\hat{K}}_{a}\left(t|X_{i}\right)}+\frac{S_{i}I\left(A_{i}=a\right)\left\{1-\hat{\omega }\left(X_{i}\right)\right\}}{\pi_{a}\hat{\omega }\left(X_{i}\right)}\;\;\overset{t}{\underset{0}{\int }}\frac{d{\hat{M}}_{a}\left(u|X_{i}\right)}{{\hat{K}}_{a}\left(u|X_{i}\right)}\frac{{\hat{H}}_{a}\left(t|X_{i}\right)}{{\hat{H}}_{a}\left(u|X_{i}\right)}+\left(1-S_{i}\right)p_{i}^{-1}\left\{{\hat{H}}_{a}\left(t|X_{i}\right)-{𝒮}_{a}(t)\right\}].$$

Based on this expansion and Central Limit Theorem, the approximate variance estimator for ${\hat{\delta }}^{\text{DR1}}(t)={\hat{𝒮}}_{1}^{\text{DR1}}(t)-{\hat{𝒮}}_{0}^{DR1}(t)$ takes the form of the below equation:

(12) $$\text{Var}\left\{{\hat{\delta }}^{\text{DR}1}(t)\right\}=\frac{1}{{\left\{\overset{n+m}{\underset{i=1}{\sum }}\left(1-S_{i}\right)p_{i}^{-1}\right\}}^{2}}\overset{n+m}{\underset{i=1}{\sum }}{\left\{{\hat{I}}_{i,1}^{\text{DR}1}(t)-{\hat{I}}_{i,0}^{\text{DR}1}(t)-{\hat{\delta }}^{\text{DR}1}(t)\right\}}^{2},$$

where

$${\hat{I}}_{i,a}^{\text{DR}1}(t)=\frac{S_{i}I\left(A_{i}=a\right)\left\{1-\hat{\omega }\left(X_{i}\right)\right\}\left\{I\left(U_{i}\geq t\right)-{\hat{K}}_{a}\left(t|X_{i}\right){\hat{H}}_{a}\left(t|X_{i}\right)\right\}}{\pi_{a}\hat{\omega }\left(X_{i}\right){\hat{K}}_{a}\left(t|X_{i}\right)}+\frac{S_{i}I\left(A_{i}=a\right)\left\{1-\hat{\omega }\left(X_{i}\right)\right\}}{\pi_{a}\hat{\omega }\left(X_{i}\right)}\overset{t}{\underset{0}{\int }}\frac{d{\hat{M}}_{a}\left(u|X_{i}\right)}{{\hat{K}}_{a}\left(u|X_{i}\right)}\frac{{\hat{H}}_{a}\left(t|X_{i}\right)}{{\hat{H}}_{a}\left(u|X_{i}\right)}\;\;+\left(1-S_{i}\right)p_{i}^{-1}\left\{{\hat{H}}_{a}\left(t|X_{i}\right)-{\hat{𝒮}}_{a}^{\text{DR}1}(t)\right\}.$$

For the DR2 estimator, we first represent this estimator as two parts, that is, ${\hat{𝒮}}_{a}^{DR2}={\hat{\nu }}_{1a}(t)+{\hat{\nu }}_{2a}(t)$, where

$${\hat{\nu }}_{1a}(t)=\overset{n+m}{\underset{i=1}{\sum }}\frac{S_{i}I\left(A_{i}=a\right)\left\{1-\hat{\omega }\left(X_{i}\right)\right\}\left\{I\left(U_{i}\geq t\right)-{\hat{K}}_{a}\left(t|X_{i}\right){\hat{H}}_{a}\left(t|X_{i}\right)\right\}/\left(\hat{\omega }\left(X_{i}\right){\hat{K}}_{a}\left(t|X_{i}\right)\right)}{\overset{n+m}{\underset{i=1}{\sum }}S_{i}I\left(A_{i}=a\right)\left\{1-\hat{\omega }\left(X_{i}\right)\right\}/\hat{\omega }\left(X_{i}\right)}\;\;+\overset{n+m}{\underset{i=1}{\sum }}\frac{S_{i}I\left(A_{i}=a\right)\left\{1-\hat{\omega }\left(X_{i}\right)\right\}/\hat{\omega }\left(X_{i}\right)}{\overset{n+m}{\underset{i=1}{\sum }}S_{i}I\left(A_{i}=a\right)\left\{1-\hat{\omega }\left(X_{i}\right)\right\}/\hat{\omega }\left(X_{i}\right)}\overset{t}{\underset{0}{\int }}\frac{d{\hat{M}}_{a}\left(u|X_{i}\right)}{{\hat{K}}_{a}\left(u|X_{i}\right)}\frac{{\hat{H}}_{a}\left(t|X_{i}\right)}{{\hat{H}}_{a}\left(u|X_{i}\right)},\;\;{\hat{\nu }}_{2a}(t)=\frac{\overset{n+m}{\underset{i=1}{\sum }}\left(1-S_{i}\right)p_{i}^{-1}{\hat{H}}_{a}\left(t|X_{i}\right)}{\overset{n+m}{\underset{i=1}{\sum }}\left(1-S_{i}\right)p_{i}^{-1}}.$$

Here, ${\hat{\nu }}_{1a}(t)$ is mostly an IPW estimator whereas ${\hat{\nu }}_{2a}(t)$ concerns a direct outcome regression estimator, and we separately quantify their variability to approximate the variability of the DR2 estimator (see Web Appendix A. 3 for additional details). From these steps, an approximate variance estimator for ${\hat{\delta }}^{DR2}(t)={\hat{𝒮}}_{1}^{DR2}(t)-{\hat{𝒮}}_{0}^{DR2}(t)$ takes the below form:

(13) $$\text{Var}\left\{{\hat{\delta }}^{\text{DR}2}(t)\right\}\approx \overset{n+m}{\underset{i=1}{\sum }}{\left\{{\hat{I}}_{i,1}^{\text{DR}2}(t)\right\}}^{2}+\overset{n+m}{\underset{i=1}{\sum }}{\left\{{\hat{I}}_{i,0}^{\text{DR}2}(t)\right\}}^{2},$$

where

$${\hat{I}}_{i,a}^{\text{DR}2}(t)={\left[\overset{n+m}{\underset{i=1}{\sum }}\frac{S_{i}I\left(A_{i}=a\right)\left\{1-\hat{\omega }\left(X_{i}\right)\right\}}{\hat{\omega }\left(X_{i}\right)}\right]}^{-1}\;\;\left[\frac{S_{i}I\left(A_{i}=a\right)\left\{1-\hat{\omega }\left(X_{i}\right)\right\}}{\hat{\omega }\left(X_{i}\right)}\left\{\frac{I\left(U_{i}\geq t\right)-{\hat{K}}_{a}\left(t|X_{i}\right){\hat{H}}_{a}\left(t|X_{i}\right)}{{\hat{K}}_{a}\left(t|X_{i}\right)}+\overset{t}{\underset{0}{\int }}\frac{d{\hat{M}}_{a}\left(u|X_{i}\right)}{{\hat{K}}_{a}\left(u|X_{i}\right)}\frac{{\hat{H}}_{a}\left(t|X_{i}\right)}{{\hat{H}}_{a}\left(u|X_{i}\right)}-{\hat{\nu }}_{1a}(t)\right\}\right]\;\;+\frac{\left(1-S_{i}\right)p_{i}^{-1}\left\{{\hat{H}}_{a}\left(t|X_{i}\right)-{\hat{\nu }}_{2a}(t)\right\}}{\sum_{i=1}^{n+m}\left(1-S_{i}\right)p_{i}^{-1}}.$$

## 5 | SIMULATION STUDY

### 5.1 | Simulation setup

In this Section, we conduct a simulation study to evaluate the finite-sample performance of the proposed estimators for TASTE, that is, δ(t). We consider a nonnested design with a RCT and an external target population (the combined sample size from the RCT and the external target population is *n* + *N* = 1 million); we further assume that the observed *m* nonrandomized individuals come from a complex survey within the target population of size *N*. For each simulation data, we first generate a baseline covariate vector $X_{i}={\left(X_{i1},X_{i2},X_{i3}\right)}^{T}$, where both *X*_*i*1_ and *X*_*i*2_ independently follow standard normal distribution *N*(0, 1), and *X*_*i*3_ ~ Bernoulli(0.5). We assume the true probability of belonging to the trial sample as follows:

$$P\left(S_{i}=1|X_{i}\right)=\frac{\text{exp}\left(\theta_{0}+\theta_{1}X_{i1}+\theta_{2}X_{i2}+\theta_{3}X_{i3}\right)}{1+\text{exp}\left(\theta_{0}+\theta_{1}X_{i1}+\theta_{2}X_{i2}+\theta_{3}X_{i3}\right)}.$$

The indicator *S* is generated as Bernoulli with probability $P\left(S_{i}=1|X_{i}\right)$ and only those with *S_i_* = 1 belong to the trial sample, and the sample size of trial is $n=\sum_{i=1}^{n+N}S_{i}$. Since the trial participants and the target population are two disjoint sets, our set up mimics a nonnested design. For those *S_i_* = 0, we consider a second logistic model to choose nonrandomized individuals resembling a survey sampling design, that is, we generate *D_i_* from a Bernoulli distribution with probability,

$$P\left(D_{i}=1|X_{i},S_{i}=0\right)=\frac{exp\left(\xi_{0}+\xi_{1}X_{i1}+\xi_{2}X_{i2}+\xi_{3}X_{i3}\right)}{1+exp\left(\xi_{0}+\xi_{1}X_{i1}+\xi_{2}X_{i2}+\xi_{3}X_{i3}\right)},$$

and include all individuals with *D* = 1 as a complex survey. Thus, the corresponding known survey weights are $p_{i}=1/P\left(D_{i}=1|X_{i}\right)$, and the survey weights for all trial randomized participants are set to be 1. Among the trial participants, the treatment indicator is randomized with *A_i_* ~ Bernoulli(0:5). In addition, under treatment status *A_i_* = 1 and *A_i_* = 0, we generate the potential failure times *T*^(*a*)^ from the following Cox models, that is,

$$\lambda_{1}\left(t|X_{i}\right)=\text{exp}\left(0.2X_{i1}-0.6X_{i2}+0.6X_{i3}\right),$$

$$\lambda_{0}\left(t|X_{i}\right)=\text{exp}\left(0.2X_{i1}+0.6X_{i2}-0.6X_{i3}\right).$$

Under the above potential outcome and censoring time models, **X**_*i*_ is clearly a set of effect modifiers that are also affecting the selection into the trial.

**FIGURE 1:**
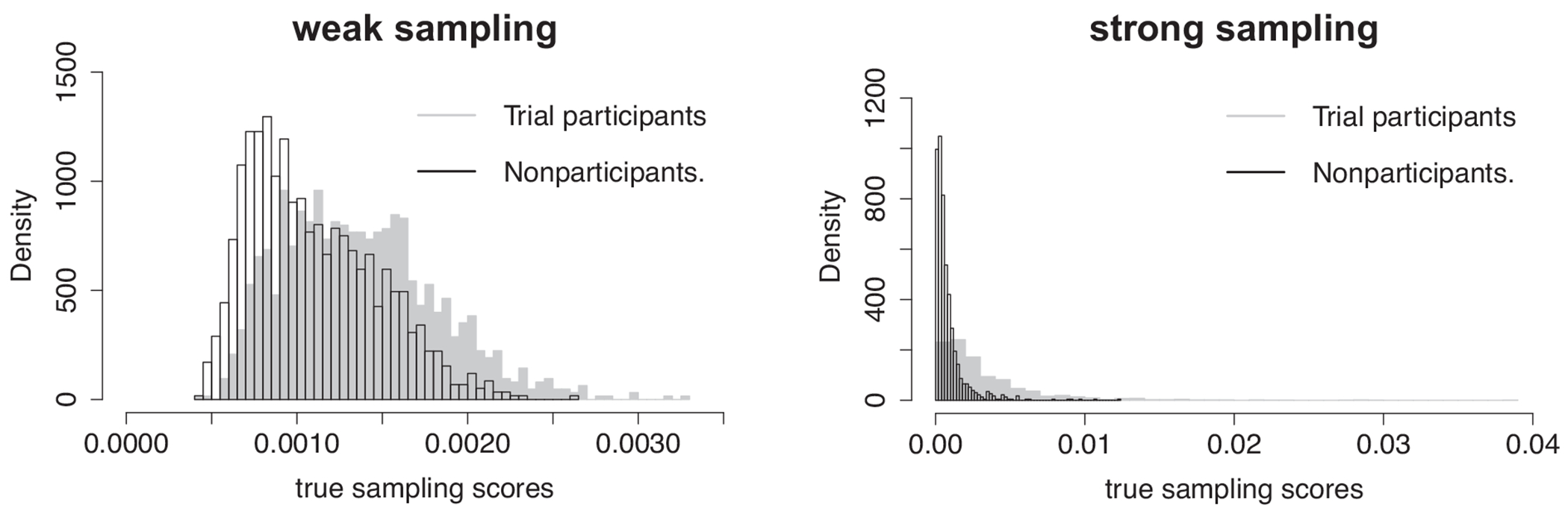
Distributions of true sampling scores associated with two different values of ***θ*** in our data generating process. Left panel: weak sampling; Right panel: strong sampling.

We consider two different values of the sampling score model parameters $\theta ={\left(\theta_{0},\theta_{1},\theta_{2},\theta_{3}\right)}^{T}$, and choose $\theta ={\left(-6.5,-0.2,-0.1,-0.5\right)}^{T}$ and $\theta ={\left(-6.7,-0.8,-0.4,-1\right)}^{T}$ to represent weak and strong selection effect in trial participation (referred to as weak and strong sampling). Figure 1 summarizes the weak and strong sampling score distributions, and we confirm that there is substantial nonoverlap between the trial sample and nonparticipants under strong sampling scenario. We choose $\xi ={\left(\xi_{0},\xi_{1},\xi_{2},\xi_{3}\right)}^{T}=(-7,0.3,0.4,0.2{)}^{T}$ for the logistic model of nonrandomized individuals. For both weak and strong sampling scenarios, our choice of simulation parameters ensures that the size of trial sample and nonparticipants are approximately n≈1200 and m≈1100. Finally, since survival outcomes are typically right-censored either due to dropout or end of follow-up, we simulate potential censoring times $C_{i}^{\left(a\right)}$ from the following Cox model,

(14) $$\lambda_{1}^{C}\left(t|X_{i},S_{i}=1\right)=\lambda_{0}^{C}\left(t|X_{i},S_{i}=1\right)=exp\left(0.1X_{i1}+0.4X_{i2}-0.6X_{i3}\right).$$

Under this model, the censoring time distribution is set to be the same for treatment and control groups and the overall censoring rate is approximately 45%. In addition to model Equation (14), which corresponds to covariate-dependent censoring, we also consider a less challenging scenario under random censoring case (i.e., censoring mechanism is independent of covariates) and generate potential censoring time from Uniform distribution, that is, $C_{i}^{\left(a\right)}|\left\{S_{i}=1\right\}∼\text{Uniform}(0,2.1)$, resulting in about 45% censoring rate. To summarize, we design simulations to reflect four typical scenarios: (1) weak sampling and covariate-dependent censoring; (2) strong sampling and covariate-dependent censoring; (3) weak sampling and random censoring; and (4) strong sampling with random censoring.

Under each simulation scenario, we consider IPW and DR estimators in settings where the required assumptions hold and when they fail to hold, as well as the weighted Kaplan–Meier estimator (WKM) as a reference estimator or competitor. The WKM estimator has been used in generalizability analysis with the inverse probability of sampling weights, ^19^ and Berkowitz et al. ^18^ considered the inverse odds of sampling weights for transportablity analysis with WKM, which we use in our comparison. Specifically, we consider the default version of the WKM estimator which assumes random censoring and therefore no adjustment for covariate-dependent censoring. This standard estimator is expected to be biased for transportability analysis when censoring depends on covariates. We also use the robust sandwich variance estimator to quantify its uncertainty, assuming that the weights are known. This estimator is implemented via the svykm function in the survey R package. For each method, to estimate the sampling score, the combined trial and survey sample is used to fit a survey weighted logistic model with $S_{i}$ as the outcome^35^ with weight $p_{i}$. The correct sampling score model is fitted using covariates ( $X_{i1},X_{i2},X_{i3}$) while the incorrect model is fitted using $X_{i1}$ as an illustration. For the outcome (i.e., survival time) $T_{i}|\left\{S_{i}=1,X_{i}\right\}$ model, the correct outcome model is fitted by Cox model with ($X_{i1},X_{i2},X_{i3}$), whereas the incorrect outcome model is fitted using the lognormal accelerated failure time (AFT) model with ($X_{i1},X_{i2},X_{i3}$ ). For the censoring $C_{i}|\left\{S_{i}=1,X_{i}\right\}$ model, the correct model is fitted using ($X_{i1},X_{i2},X_{i3}$) whereas the incorrect model is fitted using only $X_{i1}$ through a Cox model.

We simulate 5000 data replications for each scenario and evaluate the performances of WKM estimators, the IPW estimators and DR estimators for three chosen failure times *t* = (0:128,0:331,0:712) with corresponding true δ(t)=(−0.077,−0.141,−0.178). These target survival times roughly correspond to the 25th, 50th, and 75th percentiles of the empirical survival time distribution in the external target population. The variance and 95% confidence intervals of $\hat{\delta }(t)$ are calculated by using those Formulas (10)–(13) described in Section 4 and based on normal approximation. Finally, we report the following quantities under each scenario for $\hat{\delta }(t)$: Monte Carlo bias (Bias), Monte Carlo standard deviation of estimates (ESD), Monte Carlo average of estimated standard errors (ASE), coverage probability of the 95% Wald confidence intervals (CP). Example simulation code can be found at https://github.com/Zhiqiangcao/RCode_transportability.

### 5.2 | Simulation results

Tables 2 and 3 summarize the simulation results under our four data generating scenarios. As expected, the performance of the WKM transportability estimator largely depends on whether the censoring depends on baseline covariates, and can be severely biased under covariate-dependent censoring, especially at a later target time of interest. In addition, under strong sampling, the biases of WKM transportability estimator increase sharply when the sampling score model is misspecified. Furthermore, the coverage of the WKM interval estimator is frequently close to 100%, indicating that inference under this approach may be overly conservative. To summarize, this standard implementation in existing software package may be insufficient for accurate transportability of trial results to an external target population.

In our simulations with a relatively small trial size and moderate proportion of right censoring, Table 2 suggests that the IPW1 and IPW2 estimators often exhibit a certain degree of finite-sample bias, especially under strong sampling. This is expected because the IPW estimators are in general not robust to strong sampling where the inverse odds of sampling weights can be large. In addition, with censored survival outcomes, as we couple the inverse odds of sampling weights and the inverse probability of censoring weights, the final weights are only likely to be more extreme, hence compromising the stability of the IPW estimators in general. Similar to the observation made in Li et al.,^9^ the IPW2 estimator performs better than the IPW1 estimator under strong sampling but virtually identical under weak sampling; this latter case may be because the estimated final weights are generally more uniform. In addition, biases of the two IPW estimators are larger under strong sampling compared with weak sampling even when the sampling model and censoring model are correctly specified. When the sampling score model or the censoring model are incorrectly specified, the IPW estimator may exhibit larger bias at a later target time *t*. The coverage probability of the two IPW estimators based on the proposed sandwich variance estimators are frequently conservative, and sometimes close to 100%.

Table 2 has also demonstrated the robustness properties of the DR estimators, that is, the biases of $\hat{\delta }(t)$ from both DR1 and DR2 are negligible when sampling score model (for *S*) and censoring model (for *C*) are correct or the failure time outcome model (for *T*) is correct, but not necessarily both. Furthermore, under partial model correct specification, the empirical coverage probability of DR1 and DR2 estimators are generally close to the nominal 95% level. We observe that the biases from two DR estimators are more or less the same under weak and strong sampling scenarios, so that the sampling weights do not appear to have strong impact on the bias of the two DR estimators (whereas the WKM and IPW estimators are very sensitive to the distribution of sampling weights). For the DR estimator, the failure time outcome model *T* seems to have a more dominating role than the sampling score model *S* and the censoring model *C*; this is because biases are slightly larger when the outcome model is misspecified and other two models are correct than when the failure time model T is correct and other two models are misspecified. When the three working models (*T, S*, and *C*) are all incorrectly specified, it can be seen that biases of DR1 and DR2 estimators are not as large as WKM or IPW estimators when the corresponding models are incorrectly specified. Finally, under both weak and strong sampling, the two DR estimators are generally more efficient than the WKM estimators and two IPW estimators; the DR2 estimator is slightly more efficient than the DR1 estimator under strong sampling whereas their performance is similar under weak sampling, an observation that resembles that in Li et al.^9^ for generalization trial results based on noncensored outcomes.

Under random censoring and weak sampling, Table 3 shows that bias of the WKM estimator is slightly larger than two DR estimators and the coverage of the former is consistently greater than 95%. In this case, the inverse odds of sampling weights are relatively small so that the performance of the two IPW estimators depend largely on the magnitude of censoring weights. It should be noted that true censoring model is uniform in the data generating process, but we use Cox model based on (*X*_1_,*X*_2_,*X*_3_) and *X*_1_ as the “correct censoring model” and “incorrect censoring model” to estimate the inverse probability of censoring weights, so for two IPW estimators, the estimation of censoring model ${\hat{K}}_{a}\left(t|X_{i}\right)$ can be quite noisy and inefficient, leading to relatively poor performance of the IPW estimators. The same observations apply to the strong sampling scenario. However, whereas the biases of the WKM estimator increase under strong sampling, and biases from two DR estimators are comparable to those under weak sampling. Although performance of WKM is much improved under random censoring, the two DR estimators still perform better than WKM and the performance of DR2 is slightly better than that of DR1 under the strong sampling scenario. Overall, our simulation results favor the DR2 estimator over the other comparators for transporting survival analysis results in trials to an external target population.

In Web Appendix A.4, we have conducted additional simulations to further demonstrate the performance of different estimators. First, we consider a data generating process where the true sampling score model, the true censoring model and the true failure outcome model each includes a different subset of covariates in **X**_*i*_. The simulation results are qualitatively similar to those in our main simulations and continue to favor the DR estimators over WKM and IPW estimators. Second, we repeat the simulations in Table 2 under strong sampling and covariate-dependent censoring but now ignore the survey weights in each estimator. In other words, we set *p*_i_ = 1 for each observation in the combined sample to illustrate the consequence of not adhering to recommendations in Table 1. As a result, all estimators exhibit substantial bias and the DR estimators can be even more biased than the IPW estimators. This is not unexpected because DR estimators make heavier use of the survey weights than IPW estimators, and ignoring the survey weights can lead to a greater distortion of its point estimate. This observation highlights the need to incorporate survey weights when the covariates in the target population are observed only through a nonrandom sample of all nonparticipants.

## 6 | AN ILLUSTRATIVE APPLICATION: TRANSPORTING THE ACCORD TRIAL RESULTS TO AN EXTERNAL TARGET POPULATION

The ACCORD-BP trial enrolled a total of 10,251 type 2 diabetes patients with cardiovascular disease or at high cardiovascular disease risk aside beyond having diabetes.^38^ The original study considered a 2 × 2 factorial design with patients randomized to either a lipid-lowering study or a BP-lowering study. We focus on the BP-lowering study (i.e., ACCORD-BP trial) consisting of *n* = 4,733 patients. In the BP-lowering study, patients were randomized either to an intensive BP treatment arm or a standard BP treatment arm. Strategies for achieving the treatment goal or BP target was determined by study physicians under broad guidance, therefore we only consider the intention-to-treatment effect due to the assigned treatment. We focus on the primary outcome in ACCORD-BP, that is, a composite time-to-event outcome on the first occurrence of nonfatal myocardial infarction (MI), nonfatal stroke, or cardiovascular death, and a treatment is favored if it leads to longer survival. Patients outcome may be right censored either due to drop out or at the maximum follow-up of 7 years. As an illustration of our methodology, we consider transporting the ACCORD-BP results on the primary time-to-event outcome to the broader US population with diabetes. We follow Berkowitz et al.^18^ and use a sample of the National Health and Nutrition Examination Survey (NHANES) as a survey sample to represent the external target population. Specifically, we obtained the NHANES survey sample pooled across 2005–2014 including patients with diabetes (patient self-report of a diagnosis of diabetes, HbA1c ≥ 6:5%, or fasting plasma glucose (FPG) >126 mg/dl). Similar to Berkowitz et al.,^18^ we further restrict the NHANES sample to participants with at least 20 years of age, and excluded pregnant participants. This gives a subset of *m* = 1943 patients in the survey sample cohort, and the total survey weight adds up to *N* = 26,733,959, which reflects the approximate size of the external target population.

We use the combined ACCORD-BP trial and NHANES sample to fit a survey weighted logistic regression model to estimate the sampling scores, and create the inverse odds of sampling weights. The sampling score model includes age, gender, race/ethnicity, education, insurance status, smoking status, disease history variables (congestive heart failure, MI, stroke), years of diabetes, BMI, systolic and diastolic blood pressure (SBP and DBP), fasting plasma glucose (FPG), hemoglobin A1c (HBA1c), high-density lipoprotein cholesterol (HDL), low-density lipoprotein cholesterol (LDL), triglycerides (linear and squared term), estimated glomerular filtration rate (eGFR), urine albumin to creatinine ratio (UACR), interactions between race/ethnicity and BMI, as well as an interaction between years of diabetes and SBP. These two interaction terms are included because they can substantially improve the weighted covariate balance between the trial and target population; Web Figure S1 presents the standardized mean differences before and after inverse odds of sampling weighting, and demonstrate the importance of including these interaction terms in the sampling score model. To avoid the undue influence of extreme sampling scores, we truncate the sampling score at the 10% percentile of the sampling score distribution. We pursue this truncation threshold as it provides the best weighted covariate balance compared with several other thresholds lower than 10% we have considered. For the censoring model, we fit a Cox regression model separately in each treatment group among the ACCORD-BP trial sample and retained all covariates in the sampling score model but via linear terms without interactions. Our simulations suggest that DR2 has the most robust performance across scenarios, we focus on the DR2 estimator and further fit a Cox model for the failure time outcome in each treatment group with the same set of covariates. Intuitively, the outcome model can adjust for residual imbalance in covariates that are not able to be well balanced by inverse odds of sampling weights, and has potential to improve estimation efficiency.

**FIGURE 2:**
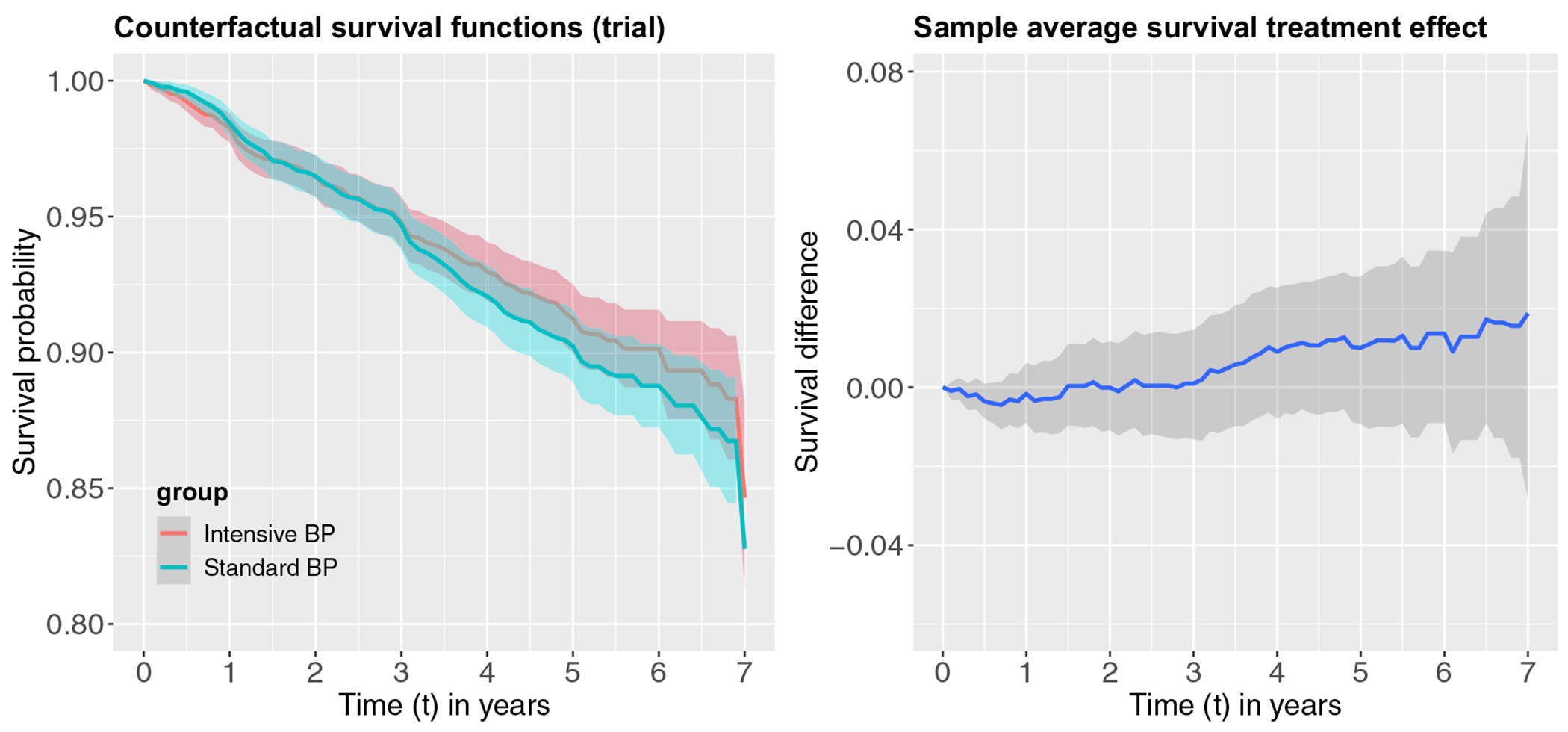
Estimated counterfactual survival functions and the sample average survival treatment effect in the ACCORD-BP trial.

**FIGURE 3:**
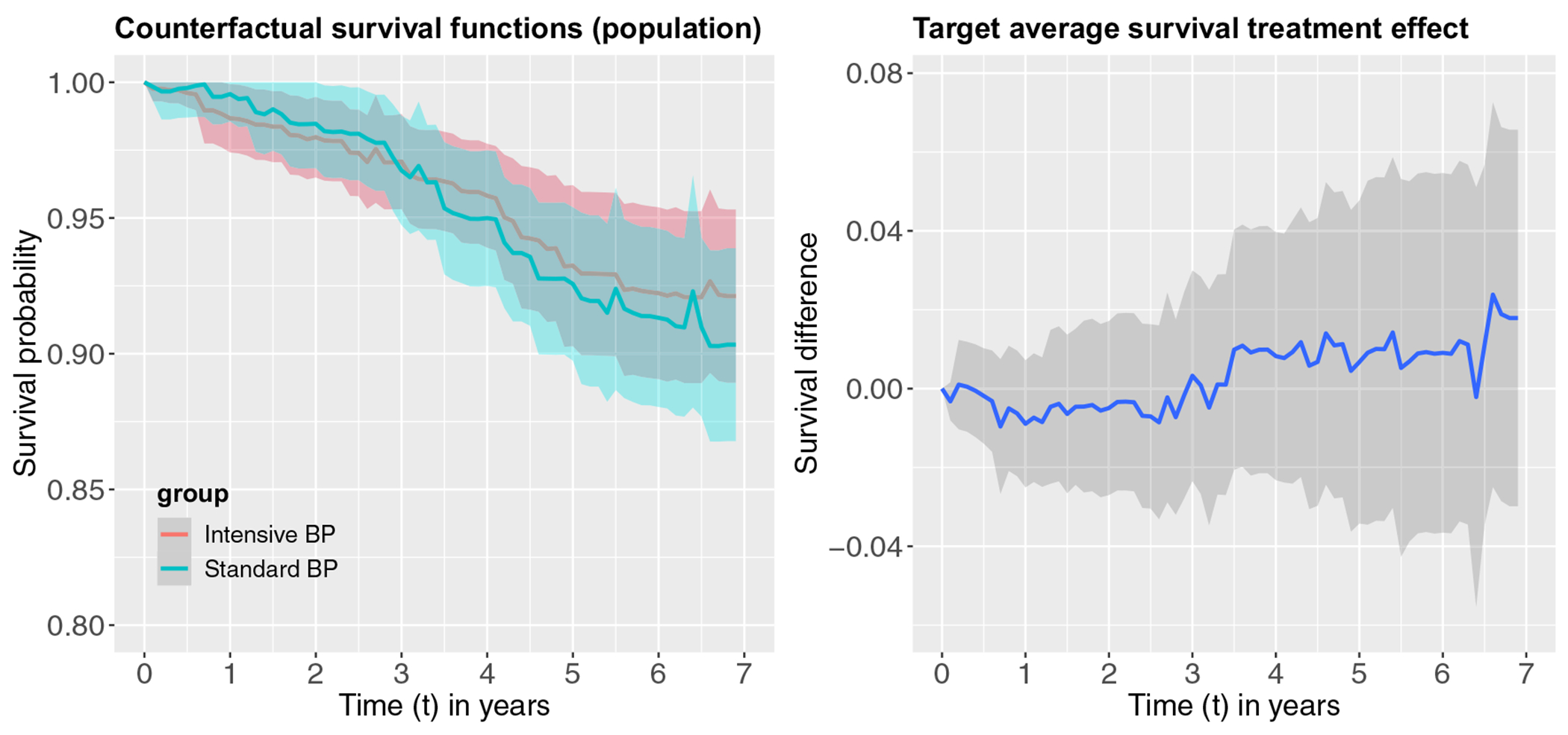
Estimated counterfactual survival functions and the target average survival treatment effect in the target population of all US populations based on the DR2 estimator.

Figure 2 presents the estimated counterfactual survival functions and the sample average survival treatment effect (SASTE) in the ACCORD-BP trial within the 7 year follow-up time. In the ACCORD-BP trial, we use a simple Kaplan–Meier estimator to estimate the counterfactual survival function and SASTE. We observe that counterfactual survival functions crossed between year 2 and year 3, corresponding to a long-term beneficial effect due to the intensive BP treatment. While in short term, the intensive BP treatment appears not to be beneficial (negative point estimates but the 95% confidence interval straddles zero), but the treatment effect increases in magnitude over time especially after year 3. Figure 3 depicts the target population counterfactual survival functions after weighting as well as the associated TASTE estimates. Slightly different from the results in the ACCORD-BP trial, the two counterfactual survival functions cross only at year 3 in the target population. Similar to the ACCORD-BP trial, the intensive BP treatment also appears not to be beneficial (negative point estimates but the 95% confidence interval straddles zero) prior to year 3 but leads to a long-term survival benefit post to year 3. The magnitude of the treatment effect, however, remains small for both the ACCORD-BP trial and the target population based on the primary composite endpoint, and the pointwise 95% confidence intervals all include zero and thus the treatment effects are not statistically significant at the 0.05 level. Table 4 provides a numerical summary of the treatment effect estimates in the ACCORD-BP trial and the external population by every half year, along with the confidence intervals. These numerical results complement the graphical results in Figures 2 and 3, and additional indicate that the transported TASTE estimates are frequently only slightly smaller than (but generally comparable) the sample average survival treatment effect estimates in the ACCORD-BP trial. Of note, since over 50% of the individuals are censored after year 5 in ACCORD-BP, the inverse probability of censoring weights tend to be much larger from year 6 onward, which might have contributed to the jump in the estimated TASTE at year 6 (Figure 3 and Table 4). Overall, the results in the ACCORD-BP trial based on the time to the first occurrence of MI, stroke, or cardiovascular death appear to be transportable to the broader US population with diabetes, but are slightly attenuated due to differential distributions of the effect modifiers. Finally, the confidence intervals are wider for the target population compared to those in the ACCORD-BP trial. This is expected because we do not observe any outcomes in the target population and inverse odds of sampling weights are likely to introduce additional uncertainty in the target population estimates.

## 7 | DISCUSSION

In this article, we consider transporting randomized trial results to an external target population where the primary outcome is time to event and subject to right censoring. Specifically, we describe two weighting and two DR estimators, and show that when both the sampling score model and censoring model are correctly specified, the proposed estimators are all pointwise consistent to the target counterfactual survival function. In addition, the two DR estimators leverage the information through direct failure time outcome regression and remain consistent to the target counterfactual survival functions if the outcome regression model is correctly specificed, regardless the specification of the sampling score model and censoring model. This additional robustness property was also demonstrated through simulation studies. For all estimators, we have proposed extensions to accommodate survey weights (or sometimes referred to as design weights), in a common scenario where the target population information is observed only through a survey sample. Beyond point estimation, we explore convenient, approximate variance estimators of each transportability estimator for statistical inference. Among the four estimators, our simulations have demonstrated that the DR2 estimator with bounded inverse odds of sampling weights has the best performance in terms of bias, efficiency and empirical coverage and therefore we consider the application of DR2 estimator to transport results from the ACCORD-BP trial.

With a right-censored survival outcome, the validity of our transportability estimators hinges upon several key assumptions: the strongly ignorable participation, the covariate-dependent censoring and the availability of survey weights. The first assumption is critical to ensure exchangeability between the potential failure time outcome in the randomized trial and target population given measured baseline covariates, and is typically assumed for transportability with noncensored outcomes.^4^ In general, this assumption calls for a comprehensive collection of baseline covariates in both the trial sample and the population sample (e.g., through a survey). This assumption, however, is not testable without additional outcome data in the target population^6^ and could be violated in the presence of unmeasured effect modifiers. When some potential effect modifiers are measured only in the trial but not in the target population, Nguyen et al.^26^ proposed methods to conduct sensitivity analysis conditional on assumed population-level information on the missing effect modifiers; the extension of their approach to accommodate our weighting and DR transportability estimators with survival data and methods to address unmeasured effect modifiers in both RCT and population data require additional research. The second assumption is often invoked in standard survival analysis and ensures that the observed and possibly censored survival time data can be used to recover the distribution of the latent failure time data. Violations of this assumption may occur, for example, when the censoring depends on post randomization time-varying covariates that are not measured in the trial. In some cases where longer survivors in the RCT experience shorter censoring times, sensitivity methods for nonignorable censoring have been developed^39^ and may be used to better capture the SASTE estimates under departure from Assumption 3, which may then inform the sensitivity of TASTE estimates. Methods for assessing the sensitivity of our transportability estimators under departures from Assumption 3 are beyond the scope of this article and represent an important direction for future research. As a final assumption, we assume the survey weights are either known from the original survey design or well-estimated from prior studies. This has been a common assumption in the literature for causal inference with complex survey designs, such as Ridgeway et al.,^40^ Austin et al.,^41^ Ackerman et al.,^10^ and Lee et al.^21,22^ We adopt this assumption to focus on key challenges of the transportability problem. If the survey weights are not known a priori by design, a further improvement of our estimators may require us to account for the uncertainty in the estimated weights that might have adjusted for clustering and nonresponse. However, characterizing the uncertainty of the survey weights will require additional covariate information from the nonparticipants that are not included in the survey sample (that is, those individuals for whom *D* = 0). Possible modifications of our estimators that address variability of the survey weights will require additional future research.

While we have provided new evidence for transporting trial results to estimate the target counterfactual survival functions, our current investigation is not without limitations. First, across all of our estimators, we have considered using the true treatment propensity score as the known allocation ratio, whereas Li et al.^9^ has demonstrated potential efficiency gain with an estimated treatment propensity score (as a way to remove chance of imbalance in the randomized trial^42^). They also found out that the efficiency gain due to an estimated propensity score is often much more substantial for weighting estimators compared with the DR estimators, and can be more substantial when the trial sample size is relatively limited. However, these insights have currently been restricted to noncensored outcomes and it is worthwhile to investigator to what extent that recommendation can be applicable to survival outcomes. Second, we have provided a set of variance estimators for the transportability estimators that ignore the uncertainty in estimating all nuisance parameters. This approach is computationally convenient, and our simulations confirm that they provide excellent coverage for the target estimand for the two DR estimators (and can be conservative for the two IPW estimators). A potential further improvement is to consider the nonparametric bootstrap variance estimators, which requires repeating the estimation procedure for the resampled data and will inevitably be much more computationally demanding (especially for the DR estimators that require estimating martingale integrals). In future work, it would be useful to evaluate whether the nonparametric bootstrap variance estimators can substantially improve upon our approximate variance estimators under realistic sample size configurations, in order to make practical recommendations on the optimal strategy to quantify the uncertainty of the transportability estimators with time-to-event outcomes. Third, different from the calibration weighting method that directly balances covariates in Lee et al.,^21^ we have considered directly estimating the inverse odds of sampling weights and inverse probability of censoring weights from parametric or semiparametric regression models. While these regression models are convenient to implement, we still observed some residual imbalances after inverse odds of sampling weighting in our illustrative application. Although the DR estimator has potential to further reduce these residual imbalances through direct outcome modeling, the approach considered in Lee et al.^21,22^ and Josey et al.^17^ may further improve the accuracy in estimating the TASTE estimand by enforcing weighted covariate balance, and a future comparative study to quantify the amount of improvement with different levels of sample sizes can be useful. Fourth, we have not explored data-driven methods to select covariates in the sampling score model, censoring model as well as the failure time model when using the proposed DR estimator. In practice, we retain the suggestion of prespecifying the set of adjustment variables (including effect modifiers) based on discussions with content knowledge experts, and this approach often suffices when there are only a handful of adjustment variables. In the presence of high-dimensional baseline covariates and without explicit knowledge on effect modifiers, one may adopt variable selection methods to identify a small set of adjustment variables for improved point estimation. For example, to inform the covariates included the sampling score model and censoring model, one may extend the outcome-adaptive LASSO approach^43^ (originally developed for analyzing observational data) which involves a higher penalty for including noneffect modifiers. The extent to which such a procedure is useful for transportability analyses is beyond the scope of our work and represents a promising area for future research.

## Supplementary Material

supplememt

Additional supporting information can be found online in the Supporting Information section at the end of this article.

## Figures and Tables

**TABLE 1:** A summary of components to include survey weights (*p_i_*) in causal transportability analyses for each estimator, when covariate information of the target population is only observed through a complex survey.

Estimator	Estimating sampling scores	Constructing the final weights	Constructing the outcome regression term
IPW1	√	√	Not applicable
IPW2	√	×	Not applicable
DR1	√	√	√
DR2	√	×	√

*Note:* For IPW1, $p_{i}$ is used to estimate $\omega \left(X_{i}\right)$ and construct the final weights (e.g., $1/\sum_{i=1}^{n+m}\left(1-S_{i}\right)p_{i}^{-1}$ ); for IPW2, $p_{i}$ is only used to estimate $\omega \left(X_{i}\right)$; for DR1, $p_{i}$ is used in estimating $\omega \left(X_{i}\right)$, constructing the final weights and constructing the outcome regression term e.g., $\left(1-S_{i}\right)p_{i}^{-1}{\hat{H}}_{a}\left(t|X_{i}\right)$; for DR2, $p_{i}$ is used to estimate $\omega \left(X_{i}\right)$ and construct the outcome regression term.

**TABLE 2:** Simulation results for estimating TASTE at three specific target survival times under covariate-dependent censoring.

Method	*T*	*S*	*C*	*t* = 0.128 and *δ*(*t*) = −0.077				*t* = 0.331 and *δ*(*t*) = −0.141				*t* = 0.712 and *δ*(*t*) = −0.178			
				Bias	ESD	ASE	CP	Bias	ESD	ASE	CP	Bias	ESD	ASE	CP
Weak sampling															
WKM		T		−0.41	2.3	2.6	97.5	−1.81	3.1	4.3	98.8	−4.62	3.6	7.2	99.9
		F		−0.12	2.1	2.5	97.5	−1.27	2.9	4.1	99.2	−3.93	3.3	6.8	100.0
IPW1		T	T	−2.43	9.6	11.2	99.1	−2.43	9.3	10.9	99.4	−2.30	9.0	10.2	99.4
		F	T	−2.19	10.1	11.3	99.1	−2.19	9.9	10.9	99.4	−1.62	9.7	10.3	99.4
		T	F	0.71	7.8	10.9	99.0	0.71	7.4	10.5	99.6	−3.79	7.0	10.0	99.5
		F	F	1.79	6.9	10.1	98.8	1.79	6.5	9.8	99.3	−2.16	6.0	9.2	99.6
IPW2		T	T	−2.41	8.2	10.0	99.7	−2.41	8.5	10.1	99.7	−2.27	8.6	9.8	99.5
		F	T	−2.18	9.0	10.2	99.7	−2.18	9.2	10.2	99.7	−1.62	9.4	10.0	99.6
		T	F	0.72	5.8	9.6	99.7	0.72	6.1	9.7	99.7	−3.76	6.3	9.5	99.6
		F	F	1.80	4.9	8.9	99.5	1.80	5.2	9.0	99.7	−2.16	5.4	8.8	99.8
DR1	T	T	T	0.00	2.2	2.2	95.2	0.03	3.0	3.0	95.5	0.01	3.5	3.5	95.0
	T	F	F	0.01	2.2	2.1	94.9	0.04	2.9	2.9	94.9	0.02	3.4	3.4	94.7
	F	T	T	−0.01	2.2	2.3	95.8	0.03	3.0	3.0	95.7	0.03	3.5	3.5	94.6
	F	F	F	0.00	2.2	2.2	95.2	−0.19	2.9	2.9	94.9	−1.33	3.4	3.3	92.6
DR2	T	T	T	0.00	2.2	2.2	95.0	0.03	3.0	2.9	94.8	0.01	3.5	3.4	94.3
	T	F	F	0.01	2.2	2.1	94.6	0.04	2.9	2.8	94.4	0.02	3.4	3.2	93.7
	F	T	T	−0.01	2.2	2.2	95.2	0.03	3.0	2.9	94.7	0.03	3.5	3.4	94.0
	F	F	F	0.01	2.2	2.1	94.5	−0.19	2.9	2.8	94.0	−1.32	3.4	3.2	92.0

Strong sampling															
WKM		T		−0.16	4.1	4.2	96.3	−1.27	5.7	7.0	98.5	−3.86	6.5	11.2	99.8
		F		−3.05	3.0	3.5	91.4	−6.39	3.9	5.8	91.4	−10.02	4.3	9.3	98.1
IPW1		T	T	−3.50	18.6	18.0	98.0	−3.50	18.1	17.1	98.6	−3.13	17.4	15.6	98.4
		F	T	3.99	15.2	14.5	99.0	−3.99	14.9	13.9	98.3	−7.23	14.4	13.0	97.4
		T	F	−0.25	16.1	17.0	98.3	−0.25	15.5	16.1	98.4	−4.53	14.7	14.7	98.0
		F	F	−1.02	11.7	13.8	98.9	−1.02	11.3	13.2	99.3	−8.44	10.7	12.3	97.9
IPW2		T	T	−3.08	15.0	14.8	99.0	−3.08	15.4	14.9	98.5	−2.35	15.6	14.2	98.3
		F	T	−3.88	13.5	12.8	99.5	−3.88	13.8	12.8	98.5	−6.99	13.8	12.4	97.1
		T	F	0.01	12.3	13.8	99.0	0.01	12.9	13.9	98.7	−3.68	13.0	13.3	97.4
		F	F	−0.93	9.5	11.9	99.6	−0.93	9.8	12.0	99.7	−8.18	10.0	11.6	97.7
DR1	T	T	T	−0.02	4.1	4.0	95.6	0.08	5.3	5.3	95.4	0.12	6.1	6.0	94.9
	T	F	F	0.02	3.1	3.0	94.4	0.07	4.1	3.9	93.3	0.06	4.7	4.3	92.4
	F	T	T	−0.06	4.2	4.0	95.7	0.10	5.4	5.3	95.4	0.25	6.3	6.1	94.6
	F	F	F	0.65	3.2	3.0	93.3	−0.21	4.2	3.9	92.7	−2.18	4.7	4.3	89.4
DR2	T	T	T	−0.02	3.8	3.6	94.7	0.08	5.0	4.9	94.5	0.11	5.8	5.5	94.0
	T	F	F	0.03	3.0	2.9	94.2	0.07	4.1	3.8	92.8	0.06	4.7	4.2	93.4
	F	T	T	−0.10	3.8	3.7	94.8	0.08	5.1	4.9	94.4	0.25	5.9	5.6	93.9
	F	F	F	0.64	3.2	2.9	92.5	−0.21	4.2	3.8	92.1	−2.14	4.6	4.2	88.6

*Note*: The columns for *T*, *S*, and *C* indicate whether the model for *T*, *S*, or *C*, is correctly (T) or incorrectly (F) specified. All values under Bias, ESD, ASE, and CP columns are multiplied by 100.

**TABLE 3:** Simulation results for estimating TASTE at three specific target survival times under random censoring.

Method	*T*	*S*	*C*	*t* = 0.128 and *δ*(*t*) = −0.077				*t* = 0.331 and *δ*(*t*) = −0.141				*t* = 0.712 and *δ*(*t*) = −0.178			
				Bias	ESD	ASE	CP	Bias	ESD	ASE	CP	Bias	ESD	ASE	CP
Weak sampling															
WKM		T		0.03	2.2	2.5	97.5	0.11	3.0	4.1	99.2	0.17	3.5	6.4	100.0
		F		0.32	2.1	2.4	96.9	0.65	2.8	3.9	99.2	0.86	3.2	6.1	99.9
IPW1		T	T	13.24	7.2	7.8	57.0	13.24	6.7	7.3	50.4	13.36	6.2	6.5	39.5
		F	T	13.02	6.8	7.5	55.8	13.02	6.4	7.0	47.4	13.56	5.9	6.3	35.9
		T	F	13.23	7.1	7.7	56.7	13.23	6.7	7.2	50.8	13.35	6.1	6.4	39.7
		F	F	13.03	6.7	7.4	55.8	13.03	6.3	7.0	47.3	13.57	5.8	6.2	36.3
IPW2		T	T	13.24	5.9	6.6	41.0	13.24	6.0	6.6	41.1	13.36	5.9	6.2	36.2
		F	T	13.02	5.6	6.4	40.2	13.02	5.7	6.4	38.4	13.56	5.6	6.0	31.9
		T	F	13.22	5.9	6.5	41.3	13.22	5.9	6.5	41.0	13.35	5.9	6.2	35.7
		F	F	13.03	5.5	6.3	39.3	13.03	5.6	6.3	38.3	13.57	5.5	6.0	32.3
DR1	T	T	T	0.01	2.2	2.2	95.3	0.02	2.9	2.9	95.4	0.02	3.3	3.3	95.0
	T	F	F	0.01	2.1	2.1	95.1	0.03	2.8	2.8	95.2	0.01	3.2	3.2	94.5
	F	T	T	−0.01	2.2	2.3	95.8	0.02	2.9	2.9	95.3	0.03	3.3	3.2	94.7
	F	F	F	−0.09	2.2	2.2	95.1	0.01	2.9	2.9	95.1	0.13	3.2	3.1	94.6
DR2	T	T	T	0.01	2.2	2.2	95.0	0.02	2.9	2.9	94.7	0.02	3.3	3.2	94.1
	T	F	F	0.01	2.1	2.1	94.8	0.03	2.8	2.8	94.5	0.01	3.2	3.1	93.5
	F	T	T	−0.01	2.2	2.2	95.2	0.03	3.0	2.9	94.7	0.03	3.5	3.4	94.0
	F	F	F	−0.01	2.2	2.2	95.3	0.02	2.9	2.9	94.8	0.03	3.3	3.1	93.8

Strong sampling															
WKM		T		0.27	4.1	4.2	96.4	0.67	5.6	6.7	98.0	0.86	6.4	10.3	99.8
		F		−2.66	3.0	3.5	93.5	−4.68	3.9	5.6	96.0	−5.84	4.1	8.6	99.8
IPW1		T	T	12.97	13.7	13.0	78.7	12.97	12.5	11.9	73.8	13.16	11.2	10.2	63.7
		F	T	15.09	9.6	9.9	60.7	15.09	9.0	9.1	63.7	11.75	8.1	8.0	59.1
		T	F	13.02	13.7	13.0	78.2	13.02	12.6	11.9	73.8	13.22	11.2	10.2	63.8
		F	F	15.09	9.5	9.8	60.7	15.09	8.9	9.0	64.4	11.76	8.0	7.9	59.5
IPW2		T	T	12.73	10.2	9.9	69.0	12.73	10.2	10.0	67.6	13.30	9.9	9.2	59.7
		F	T	15.03	7.8	8.1	45.1	15.03	7.9	8.1	56.0	11.82	7.7	7.6	56.0
		T	F	12.78	10.3	9.9	68.7	12.78	10.3	9.9	66.9	13.35	9.9	9.2	59.6
		F	F	15.03	7.7	8.0	45.7	15.03	7.8	8.1	55.9	11.82	7.6	7.5	56.0
DR1	T	T	T	−0.04	4.0	3.9	95.1	0.07	5.0	5.1	95.4	0.04	5.6	5.5	95.0
	T	F	F	0.03	3.0	3.0	94.0	0.06	4.0	3.8	93.8	−0.01	4.4	4.1	93.1
	F	T	T	−0.10	4.0	4.0	95.4	0.08	5.1	5.1	95.5	0.13	5.8	5.5	95.0
	F	F	F	0.65	3.2	3.0	93.0	0.04	4.0	3.8	93.4	−0.84	4.3	4.1	92.8
DR2	T	T	T	−0.04	3.7	3.6	94.4	0.07	4.7	4.7	94.7	0.03	5.2	5.0	94.1
	T	F	F	0.03	3.0	2.9	93.8	0.06	4.0	3.7	93.1	−0.01	4.3	4.0	92.3
	F	T	T	−0.12	3.7	3.6	94.6	0.06	4.7	4.7	94.7	0.14	5.3	5.1	93.9
	F	F	F	0.64	3.1	2.9	92.5	0.04	4.0	3.7	92.4	−0.82	4.3	4.0	92.2

*Note*: The columns for *T*, *S*, and *C* indicate whether the model for *T*, *S*, or *C*, is correctly (T) or incorrectly (F) specified. All values under Bias, ESD, ASE, and CP columns are multiplied by 100.

**TABLE 4:** Estimated difference in the counterfactual survival probability in the ACCORD-BP trial and the external US target population based on the DR2 estimator at selected landmark survival times with an increment of 0.5 years.

Time (in years)	ACCORD-BP trial		Target Population	
	Estimated effect (%)	95% CI (%)	Estimated TASTE (%)	95% CI (%)
0.5	−0.360	(−0.786, 0.090)	−0.186	(−1.395, 1.023)

1.0	−0.165	(−0.905, 0.618)	−0.893	(−2.505, 0.719)

1.5	0.034	(−0.964, 1.107)	−0.643	(−2.653, 1.367)

2.0	−0.009	(−1.090, 1.161)	−0.493	(−2.700, 1.714)

2.5	0.041	(−1.190, 1.373)	−0.706	(−3.044, 1.632)

3.0	0.096	(−1.321, 1.457)	0.326	(−2.346, 2.998)

3.5	0.578	(−0.973, 2.082)	0.988	(−2.057, 4.033)

4.0	0.902	(−0.802, 2.531)	0.823	(−2.322, 3.969)

4.5	1.065	(−0.693, 2.731)	0.677	(−2.972, 4.326)

5.0	1.004	(−0.930, 2.805)	0.675	(−3.422, 4.771)

5.5	1.308	(−0.923, 3.310)	0.525	(−4.263, 5.313)

6.0	1.361	(−0.904, 3.463)	0.907	(−3.653, 5.466)

6.5	1.715	(−0.905, 4.410)	1.105	(−3.460, 5.670)

*Note*: All values in the table should be divided by 100. For example, at 0.5 year, the estimated survival probability differences in the ACCORD-BP trial and target population are −0:360/100=−0:0036 and −0:186/100=−0:00186, respectively. CI stands for confidence interval.

## Data Availability

The ACCORD study data are available from the NHLBI BioLINCC website and can be requested at https://biolincc.nhlbi.nih.gov/studies/accord/. Fan Li obtained access to the publicly available ACCORD study data according to a Data Use Agreement from BioLINCC and performed the data analysis in Section 6. The NHANES data is publicly available and can be obtained from https://wwwn.cdc.gov/nchs/nhanes/. The authors are very grateful to Dr. Seth A. Berkowitz for sharing a clean version of the NHANES sample data for our illustrative application.
