# Supplementary material for: Transporting randomized trial results to estimate counterfactual survival functions in target populations
Source: Pharm Stat. Author manuscript; Available in PMC 2026 Mar 21. (PMC13005602; doi:10.1002/pst.2354)
Supplement: supplememt [file NIHMS2151148-supplement-supplememt.pdf]

Web Appendix for "Transporting randomized trial results to estimate counterfactual survival functions in target populations"

Zhiqiang Cao[1], Youngjoo Cho[2] and Fan Li[3,4]

[1]Department of Mathematics, College of Big Data and Internet, Shenzhen Technology University, Shenzhen, P. R. China

[2]Department of Applied Statistics, Konkuk University, Seoul, Republic of Korea

[3]Department of Biostatistics, Yale University School of Public Health, Connecticut, U.S.A

[4]Center for Methods in Implementation and Prevention Science, Yale School of Public Health, Connecticut, U.S.A

A.1.Consistency of IPW estimators

In the following technical exposition, we assume some standard regularity conditions for causal survival analysis, similar to those in Chen and Tsiatis (2001) and Bai et al. (2013). We define "$\xrightarrow{\mathcal{P}}$" as convergence in probability. If the model for $\omega(\mathbf{X})$ is correct, then the estimator for the sampling score model parameter, $\widehat{\boldsymbol{\theta}} \xrightarrow{\mathcal{P}} \boldsymbol{\theta}$ and therefore $\widehat{\omega}(\mathbf{X}) \xrightarrow{\mathcal{P}} \omega(\mathbf{X})$. In addition, if the model for $C|\{S=1,\mathbf{X}\}$ is correct, then the estimator for the censoring score model parameter $\widehat{\boldsymbol{\theta}} \xrightarrow{\mathcal{P}} \boldsymbol{\theta}$ and $\widehat{K}_a(t,\mathbf{X}) \xrightarrow{\mathcal{P}} K_a(t,\mathbf{X})$. Similarly, if the model for $T|\{S=1,\mathbf{X}\}$ is correct, then we have $\widehat{H}_a(t,\mathbf{X}) \xrightarrow{\mathcal{P}} H_a(t,\mathbf{X})$. In more general cases allowing for model misspecification, we will have $\widehat{\omega}(\mathbf{X}) \xrightarrow{\mathcal{P}} \omega^*(\mathbf{X})$, $\widehat{K}_a(t,\mathbf{X}) \xrightarrow{\mathcal{P}} K_a^*(t,\mathbf{X})$ and $\widehat{H}_a(t,\mathbf{X}) \xrightarrow{\mathcal{P}} H_a^*(t,\mathbf{X})$, where $\omega^*(\mathbf{X})$, $K_a^*(t,\mathbf{X})$ and $H_a^*(t,\mathbf{X})$ denote the sampling score, censoring score and outcome survival function evaluated under the limit of parameter under model misspecification (White, 1982), which may or may not be equal to the corresponding truth (or data generating process); notice that we use '$*$' to represent the misspecified asymptotic limit. For simplicity, we focus on the estimators in Section 3.2 and Section 3.3, where the covariate information for the target population is fully observed (in other words, $N=m$, so the number of total observations is $N^{tot}=n+m=n+N$). Extensions to survey designs are straightforward and therefore omitted.

For the IPW1 estimator, when both sampling score model and censoring model are correctly

specified, we have

$$
\begin{aligned}
\widehat{\mathcal{S}}_a^{\mathrm{IPW1}}(t) =& \frac{N^{tot}}{m} \times \frac{1}{N^{tot}} \sum_{i=1}^{N^{tot}} \frac{\Delta_i S_i I(A_i = a)\{1-\widehat{\omega}(\mathbf{X}_i)\} I(U_i \geq t)}{\widehat{\omega}(\mathbf{X}_i)\pi_a \widehat{K}_a(U_i|\mathbf{X}_i)} \\
\xrightarrow{\mathcal{P}}& \frac{1}{P(S=0)} E\left[\frac{SI(A=a)\{1-\omega(\mathbf{X})\}I(C^{(a)} \geq t, C^{(a)} \geq T^{(a)})I(T^{(a)} \geq t)}{\pi_a \omega(\mathbf{X}) K_a(U|\mathbf{X})}\right] \\
=& \frac{P(S=1)}{P(S=0)} E\left[\frac{I(A=a)\{1-\omega(\mathbf{X})\}I(C^{(a)} \geq T^{(a)})}{\pi_a \omega(\mathbf{X}) K_a(T^{(a)}|\mathbf{X})} I(T^{(a)} \geq t)|S=1\right] \\
=& \frac{P(S=1)}{P(S=0)} E\left[\frac{\{1-\omega(\mathbf{X})\}I(C^{(a)} \geq T^{(a)})}{\omega(\mathbf{X}) K_a(T^{(a)}|\mathbf{X})} I(T^{(a)} \geq t)|S=1\right] \\
=& \frac{1}{P(S=0)} E\left[E\left[\frac{S\{1-\omega(\mathbf{X})\}I(C^{(a)} \geq T^{(a)})}{\omega(\mathbf{X}) K_a(T^{(a)}|\mathbf{X})} I(T^{(a)} \geq t)|\mathbf{X}\right]\right] \\
=& \frac{1}{P(S=0)} E\left[E\left[\{1-\omega(\mathbf{X})\}I(T^{(a)} \geq t)|\mathbf{X}\right]\right] = \frac{E[(1-S)I(T^{(a)} \geq t)]}{P(S=0)} = \mathcal{S}_a(t),
\end{aligned}
$$

where the third equality is due to Assumption 1 (*Randomization*), the fifth equality is due to Assumption 2 (*Strongly Ignorable Participation*) and Assumption 3 (*Covariate-Dependent Censoring*), and the sixth equality is based on law of total probability. Similarly, we can show $\widehat{\mathcal{S}}_a^{\mathrm{IPW2}}(t)$ is consistent under correctly specified sampling score model and censoring model. In the presence of a survey design with known survey weights, the IPW estimators in Section 3.4 are consistent based on the identity $E[(1-S_i)p_i^{-1}] = P(S_i = 0)$ (which is the basis of the normalization constant for the IPW1 estimator in Section 3.4), and the rest follows the above derivation.

A.2. Sketch of Proof on double robustness property for DR estimators

We focus on showing the double robustness property of $\widehat{\mathcal{S}}_a^{\mathrm{DR1}}(t)$, as the steps for showing the property for $\widehat{\mathcal{S}}_a^{\mathrm{DR2}}(t)$ is completely analogous. To proceed, we need to find the following expectation:

$$
\begin{aligned}
& E\Bigg[\frac{SI(A=a)\{1-\omega^*(\mathbf{X})\}\{I(U \geq t) - K_a^*(t|\mathbf{X})H_a^*(t|\mathbf{X})\}}{\omega^*(\mathbf{X})\pi_a K_a^*(t|\mathbf{X})} \\
& + \frac{SI(A=a)\{1-\omega^*(\mathbf{X})\}}{\pi_a \omega^*(\mathbf{X})} \int_0^t \frac{dM_a^*(u|\mathbf{X})}{K_a^*(u|X)} \frac{H_a^*(t|\mathbf{X})}{H_a^*(u|\mathbf{X})} + (1-S)H_a^*(t|\mathbf{X})\Bigg] \qquad \text{(A0.1)} \\
=& E\Bigg[\frac{S\{1-\omega^*(\mathbf{X})\}\{I(T^{(a)} \geq t)I(C^{(a)} \geq t) - K_a^*(t|\mathbf{X})H_a^*(t|\mathbf{X})\}}{\omega^*(\mathbf{X})K_a^*(t|\mathbf{X})} \\
& + \frac{S\{1-\omega^*(\mathbf{X})\}}{\omega^*(\mathbf{X})} \int_0^t \frac{dM_a^*(u|\mathbf{X})}{K_a^*(u|\mathbf{X})} \frac{H_a^*(t|\mathbf{X})}{H_a^*(u|\mathbf{X})} + (1-S)H_a^*(t|\mathbf{X})\Bigg]. \qquad \text{(A0.2)}
\end{aligned}
$$

where the equality is due to SUTVA and Assumption 1, and $dM_a^*(u|\mathbf{X}) = dN_a(u) - I(U^{(a)} \geq u)\lambda_a^*(u|\mathbf{X})$ is the residual term associated with the counting process of the observed censoring time.

First, we notice the following identity

$$\int_0^t \frac{dM_a^*(u|\mathbf{X})}{K_a^*(u|\mathbf{X})} = \int_0^t \frac{I(T^{(a)} \geq u)\{d\tilde{N}_a(u) - I(C^{(a)} \geq u)\lambda_a^*(u|\mathbf{X})\}}{K_a^*(u|\mathbf{X})},$$

where $\tilde{N}_a(u) = I(C^{(a)} \leq u)$, and $dN_a(u) = I(T^{(a)} \geq u)d\tilde{N}_a(u)$. Since $\lambda_a(u|\mathbf{X}) = -d\log K_a(u|\mathbf{X})/du$, we have

$$\int_0^t \frac{d\lambda_a(u|\mathbf{X})}{K_a(u|\mathbf{X})} = \frac{1}{K_a(t|\mathbf{X})} - \frac{1}{K_a(0|\mathbf{X})} = \frac{1}{K_a(t|\mathbf{X})} - 1.$$

Define $d\tilde{M}_a^*(u|\mathbf{X}) = d\tilde{N}_a(u) - I(C^{(a)} \geq u)\lambda_a^*(u|\mathbf{X})$ as residual term associated with the counting process for the latent censoring time. Then, we have

$$\begin{aligned}
\int_0^t \frac{d\tilde{M}_a^*(u|\mathbf{X})}{K_a^*(u|\mathbf{X})} &= \int_0^t \frac{d\tilde{N}_a(u) - I(C^{(a)} \geq u)\lambda_a^*(u|\mathbf{X})}{K_a^*(u|\mathbf{X})} = \frac{I(C^{(a)} \leq t)}{K_a^*(C^{(a)}|\mathbf{X})} - \int_0^{t\wedge C^{(a)}} \frac{d\lambda_a^*(u|\mathbf{X})}{K_a^*(u|\mathbf{X})} \\
=& \frac{I(C^{(a)} \leq t)}{K_a^*(C^{(a)}|\mathbf{X})} - I(C^{(a)} \leq t)\left[\frac{1}{K_a^*(C^{(a)}|\mathbf{X})} - 1\right] - I(C^{(a)} \geq t)\left[\frac{1}{K_a^*(t|\mathbf{X})} - 1\right] = 1 - \frac{I(C^{(a)} \geq t)}{K_a^*(t|\mathbf{X})}.
\end{aligned}$$

With these steps, we note that for the first and third terms in the expectation (A0.2), we have

$$\begin{aligned}
&\frac{S\{1-\omega^*(\mathbf{X})\}I(T^{(a)} \geq t)I(C^{(a)} \geq t)}{\omega^*(\mathbf{X})K_a^*(t|\mathbf{X})} - \frac{S\{1-\omega^*(\mathbf{X})\}}{\omega^*(\mathbf{X})}H_a^*(t|\mathbf{X}) + (1-S)H_a^*(t|\mathbf{X}) \\
=& \frac{S\{1-\omega^*(\mathbf{X})\}I(T^{(a)} \geq t)}{\omega^*(\mathbf{X})}\left[1 - \int_0^t \frac{d\tilde{M}_a^*(u|\mathbf{X})}{K_a^*(u|\mathbf{X})}\right] - \frac{S\{1-\omega^*(\mathbf{X})\}}{\omega^*(\mathbf{X})}H_a^*(t|\mathbf{X}) + (1-S)H_a^*(t|\mathbf{X}) \\
=& (1-S)I(T^{(a)} \geq t) + \frac{S\{1-\omega^*(\mathbf{X})\}I(T^{(a)} \geq t)}{\omega^*(\mathbf{X})} - (1-S)I(T^{(a)} \geq t) \\
&- \frac{S\{1-\omega^*(\mathbf{X})\}I(T^{(a)} \geq t)}{\omega^*(\mathbf{X})}\int_0^t \frac{d\tilde{M}_a^*(u|\mathbf{X})}{K_a^*(u|\mathbf{X})} - \frac{S\{1-\omega^*(\mathbf{X})\}}{\omega^*(\mathbf{X})}H_a^*(t|\mathbf{X}) + (1-S)H_a^*(t|\mathbf{X}) \\
=& (1-S)I(T^{(a)} \geq t) + \frac{S-\omega^*(\mathbf{X})}{\omega^*(\mathbf{X})}\{I(T^{(a)} \geq t) - H_a^*(t|\mathbf{X})\} - \frac{S\{1-\omega^*(\mathbf{X})\}I(T^{(a)} \geq t)}{\omega^*(\mathbf{X})}\int_0^t \frac{d\tilde{M}_a^*(u|\mathbf{X})}{K_a^*(u|\mathbf{X})}.
\end{aligned}$$

Therefore, the whole term within the expectation in (A0.2) becomes

$$\begin{aligned}
&\frac{S\{1-\omega^*(\mathbf{X})\}\{I(T^{(a)} \geq t)I(C^{(a)} \geq t) - K_a^*(t|\mathbf{X})H_a^*(t|\mathbf{X})\}}{\omega^*(\mathbf{X})K_a^*(t|\mathbf{X})} \\
&+ \frac{S\{1-\omega^*(\mathbf{X})\}}{\omega^*(\mathbf{X})}\int_0^t \frac{dM_a^*(u|\mathbf{X})}{K_a^*(u|X)}\frac{H_a^*(t|\mathbf{X})}{H_a^*(u|\mathbf{X})} + (1-S)H_a^*(t|\mathbf{X}) \qquad \text{(A0.3)} \\
=& (1-S)I(T^{(a)} \geq t) + \frac{S-\omega^*(\mathbf{X})}{\omega^*(\mathbf{X})}\{I(T^{(a)} \geq t) - H_a^*(t|\mathbf{X})\} \\
&+ \frac{S\{1-\omega^*(\mathbf{X})\}}{\omega^*(\mathbf{X})}\int_0^t \frac{d\tilde{M}_a^*(u|\mathbf{X})}{K_a^*(u|\mathbf{X})}\left[I(T^{(a)} \geq u)\frac{H_a^*(t|\mathbf{X})}{H_a^*(u|\mathbf{X})} - I(T^{(a)} \geq t)\right]. \qquad \text{(A0.4)}
\end{aligned}$$

It is therefore sufficient to study the expectation of (A0.4). Note that $E[(1-S)I(T^{(a)} \geq t)] = \mathcal{S}_a(t)$ by definition, and

$$E\left[\frac{S-\omega^*(\mathbf{X})}{\omega^*(\mathbf{X})}\{I(T^{(a)} \geq t) - H_a^*(t|\mathbf{X})\}\right] = 0$$

if either $\omega^*(\mathbf{X}) = P(S=1|\mathbf{X})$ or $H_a^*(t|\mathbf{X}) = P(T^{(a)} \geq t|\mathbf{X})$. Finally, if $K_a^*(u|\mathbf{X}) = P(C^{(a)} \geq u|\mathbf{X}) = P(C \geq u|A=a,\mathbf{X})$, then $d\tilde{M}_a^*(u|\mathbf{X}) = d\tilde{N}_a(u) - I(C^{(a)} \geq u)\lambda_a^*(u|\mathbf{X})$ is a martingale increment and therefore the third term has mean zero. Finally, if $H_a^*(t|\mathbf{X}) = P(T^{(a)} \geq t|\mathbf{X})$, then by interchanging the integration and expectation signs

$$\begin{aligned}
&E\left[\frac{S\{1-\omega^*(\mathbf{X})\}}{\omega^*(\mathbf{X})}\int_0^t \frac{d\tilde{M}_a^*(u|\mathbf{X})}{K_a^*(u|\mathbf{X})}\left\{I(T^{(a)} \geq u)\frac{H_a^*(t|\mathbf{X})}{H_a^*(u|\mathbf{X})} - I(T^{(a)} \geq t)\right\}\right] \\
&= \int_0^t E\left[\frac{S\{1-\omega^*(\mathbf{X})\}}{\omega^*(\mathbf{X})}\frac{d\tilde{M}_a^*(u|\mathbf{X})}{K_a^*(u|\mathbf{X})}\left\{I(T^{(a)} \geq u)\frac{H_a^*(t|\mathbf{X})}{H_a^*(u|\mathbf{X})} - I(T^{(a)} \geq t)\right\}\right] \\
&= \int_0^t E\left[\frac{S\{1-\omega^*(\mathbf{X})\}}{\omega^*(\mathbf{X})}\frac{d\tilde{M}_a^*(u|\mathbf{X})}{K_a^*(u|\mathbf{X})}E\left\{I(T^{(a)} \geq u)\frac{H_a^*(t|\mathbf{X})}{H_a^*(u|\mathbf{X})} - I(T^{(a)} \geq t)|S,\mathbf{X},C^{(a)}\right\}\right].
\end{aligned}$$

Under Assumption 3 (*Covariate-Dependent Censoring)*, we have

$$E\left\{I(T^{(a)} \geq u)\frac{H_a^*(t|\mathbf{X})}{H_a^*(u|\mathbf{X})} - I(T^{(a)} \geq t)|S,\mathbf{X},C^{(a)}\right\} = H_a^*(t|\mathbf{X}) - P(T^{(a)} \geq t|\mathbf{X}) = 0.$$

Therefore to summarize, the double robustness property of $\widehat{\mathcal{S}}_a^{\mathrm{DR1}}(t)$ holds because this estimator is (pointwise) consistent to $\mathcal{S}_a(t)$ when either (i) the sampling score model and censoring model are correctly specified or (ii) the survival outcome model (outcome model) is correctly specified. The double robustness property of $\widehat{\mathcal{S}}_a^{\mathrm{DR2}}(t)$ can be shown using the same arguments above and therefore are omitted. Finally, in the presence of a survey design with known survey weights, the DR estimators in Section 3.4 are consistent based on the identity $E[(1-S_i)p_i^{-1}] = P(S_i = 0)$, and the rest follows the above derivation.

A.3. Derivation of an approximate variance estimator for $\widehat{\delta}_a^{\mathbf{DR2}}(t)$

First, we let

$$\widehat{h}_{1a}(\mathbf{X}_i) = \frac{S_i I(A_i = a)\{1-\widehat{\omega}(\mathbf{X}_i)\}}{\widehat{\omega}(\mathbf{X}_i)}, \qquad \widehat{h}_{2a}(\mathbf{X}_i) = \frac{(1-S_i)p_i^{-1}}{\sum_{i=1}^{n+m}(1-S_i)p_i^{-1}},$$

and

$$
\begin{aligned}
\nu_{1a}(t) =& \frac{I(U \geq t) - K_a(t|\mathbf{X})H_a(t|\mathbf{X})}{K_a(t|\mathbf{X})} + \int_0^t \frac{dM_a(u|\mathbf{X})}{K_a(u|\mathbf{X})}\frac{H_a(t|\mathbf{X})}{H_a(u|\mathbf{X})} \\
=& \frac{I(U \geq t)}{K_a(t|\mathbf{X})} - H_a(t|\mathbf{X}) + \int_0^t \frac{dM_a(u|\mathbf{X})}{K_a(u|\mathbf{X})}\frac{H_a(t|\mathbf{X})}{H_a(u|\mathbf{X})}, \\
\nu_{2a}(t) =& H_a(t|\mathbf{X}), \\
\widehat{\nu}_{1i,a}(t) =& \frac{I(U_i \geq t) - \widehat{K}_a(t|\mathbf{X}_i)\widehat{H}_a(t|\mathbf{X}_i)}{\widehat{K}_a(t|\mathbf{X}_i)} + \int_0^t \frac{d\widehat{M}_a(u|\mathbf{X}_i)}{\widehat{K}_a(u|\mathbf{X}_i)}\frac{\widehat{H}_a(t|\mathbf{X}_i)}{\widehat{H}_a(u|\mathbf{X}_i)} \\
=& \frac{I(U_i \geq t)}{\widehat{K}_a(t|\mathbf{X}_i)} - \widehat{H}_a(t|\mathbf{X}_i) + \int_0^t \frac{d\widehat{M}_a(u|\mathbf{X}_i)}{\widehat{K}_a(u|\mathbf{X}_i)}\frac{\widehat{H}_a(t|\mathbf{X}_i)}{\widehat{H}_a(u|\mathbf{X}_i)}, \\
\widehat{\nu}_{2i,a}(t) =& \widehat{H}_a(t|\mathbf{X}_i).
\end{aligned}
$$

Then by the construction of $\widehat{\mathcal{S}}_a^{\mathrm{DR2}}(t)$,

$$
\begin{aligned}
0 =& \sum_{i=1}^{n+m} \frac{S_i I(A_i = a)\{1 - \widehat{\omega}(\mathbf{X}_i)\}/\widehat{\omega}(\mathbf{X}_i)}{\sum_{i=1}^{n+m} S_i I(A_i = a)\{1 - \widehat{\omega}(\mathbf{X}_i)\}/\widehat{\omega}(\mathbf{X}_i)}\Bigg(\frac{I(U_i \geq t) - \widehat{K}_a(t|\mathbf{X}_i)\widehat{H}_a(t|\mathbf{X}_i)}{\widehat{K}_a(t|\mathbf{X}_i)} \\
&+ \int_0^t \frac{d\widehat{M}_a(u|\mathbf{X}_i)}{\widehat{K}_a(u|\mathbf{X}_i)}\frac{\widehat{H}_a(t|\mathbf{X}_i)}{\widehat{H}_a(u|\mathbf{X}_i)} - \widehat{\nu}_{1a}(t)\Bigg) + \sum_{i=1}^{n+m} \frac{(1 - S_i)p_i^{-1}}{\sum_{i=1}^{n+m}(1 - S_i)p_i^{-1}}\{\widehat{H}_a(t|\mathbf{X}_i) - \widehat{\nu}_{2a}(t)\} \\
=& \sum_{i=1}^{n+m} \left[\frac{\widehat{h}_{1a}(\mathbf{X}_i)}{\sum_{i=1}^{n+m}\widehat{h}_{1a}(\mathbf{X}_i)}\Big(\widehat{\nu}_{1i,a}(t) - \widehat{\nu}_{1a}(t)\Big) + \frac{\widehat{h}_{2a}(\mathbf{X}_i)}{\sum_{i=1}^{n+m}\widehat{h}_{2a}(\mathbf{X}_i)}\Big(\widehat{\nu}_{2i,a}(t) - \widehat{\nu}_{2a}(t)\Big)\right] \\
=& \sum_{i=1}^{n+m} \Bigg[\frac{\widehat{h}_{1a}(\mathbf{X}_i)}{\sum_{i=1}^{n+m}\widehat{h}_{1a}(\mathbf{X}_i)}\Big\{(\widehat{\nu}_{1i,a}(t) - \nu_{1a}(t)) + (\nu_{1a}(t) - \widehat{\nu}_{1a}(t))\Big\} \\
&+ \frac{\widehat{h}_{2a}(\mathbf{X}_i)}{\sum_{i=1}^{n+m}\widehat{h}_{2a}(\mathbf{X}_i)}\Big\{(\widehat{\nu}_{2i,a}(t) - \nu_{2a}(t)) + (\nu_{2a}(t) - \widehat{\nu}_{2a}(t))\Big\}\Bigg].
\end{aligned}
$$

By consistency result proved in A.1., $\nu_{1a}(t) + \nu_{2a}(t)$ converges in probability to $\mathcal{S}_a(t)$, and $\widehat{\mathcal{S}}_a^{\mathrm{DR2}}(t)$ converges in probability to $\mathcal{S}_a(t)$. Hence our expansion of interest, $\widehat{\mathcal{S}}_a^{\mathrm{DR2}}(t) - \mathcal{S}_a(t)$ is

$$
\begin{aligned}
\widehat{\mathcal{S}}_a^{\mathrm{DR2}}(t) - \mathcal{S}_a(t) &= \widehat{\nu}_{1a}(t) + \widehat{\nu}_{2a}(t) - \mathcal{S}_a(t) \\
&\approx \sum_{i=1}^{n+m} \left[\frac{\widehat{h}_{1a}(\mathbf{X}_i)}{\sum_{i=1}^{n+m}\widehat{h}_{1a}(\mathbf{X}_i)}(\widehat{\nu}_{1i,a}(t) - \nu_{1a}(t)) + \frac{\widehat{h}_{2a}(\mathbf{X}_i)}{\sum_{i=1}^{n+m}\widehat{h}_{2a}(\mathbf{X}_i)}(\widehat{\nu}_{2i,a}(t) - \nu_{2a}(t))\right] \\
&= \frac{1}{\sum_{i=1}^{n+m}\widehat{h}_{1a}(\mathbf{X}_i)}\left(\sum_{i=1}^{n+m}\widehat{h}_{1a}(\mathbf{X}_i)\{\widehat{\nu}_{1i,a}(t) - \nu_{1a}(t)\}\right) \\
&\quad + \frac{1}{\sum_{i=1}^{n+m}\widehat{h}_{2a}(\mathbf{X}_i)}\left(\sum_{i=1}^{n+m}\widehat{h}_{2a}(\mathbf{X}_i)\{\widehat{\nu}_{2i,a}(t) - \nu_{2a}(t)\}\right).
\end{aligned}
$$

Hence the influence function for $\widehat{\mathcal{S}}_a^{\mathrm{DR2}}(t)$ is

$$\widehat{I}_{i,a}^{\mathrm{DR2}}(t) = \Bigg[\sum_{i=1}^{n+m} \frac{S_i I(A_i = a)\{1-\widehat{\omega}(\mathbf{X}_i)\}}{\widehat{\omega}(\mathbf{X}_i)}\Bigg]^{-1}\Bigg[\frac{S_i I(A_i = a)\{1-\widehat{\omega}(\mathbf{X}_i)\}}{\widehat{\omega}(\mathbf{X}_i)}\Bigg\{\frac{I(U_i \geq t) - \widehat{K}_a(t|\mathbf{X}_i)\widehat{H}_a(t|\mathbf{X}_i)}{\widehat{K}_a(t|\mathbf{X}_i)} + \int_0^t \frac{d\widehat{M}_a(u|\mathbf{X}_i)}{\widehat{K}_a(u|\mathbf{X}_i)}\frac{\widehat{H}_a(t|\mathbf{X}_i)}{\widehat{H}_a(u|\mathbf{X}_i)} - \widehat{\nu}_{1a}(t)\Bigg\}\Bigg] + \frac{(1-S_i)p_i^{-1}\{\widehat{H}_a(t|\mathbf{X}_i) - \widehat{\nu}_{2a}(t)\}}{\sum_{i=1}^{n+m}(1-S_i)p_i^{-1}}.$$

and hence the variance estimator for $\widehat{\delta}_a^{\mathrm{DR2}}(t)$ is

$$\sum_{i=1}^{n+m}\{\widehat{I}_{i,1}^{\mathrm{DR2}}(t)\}^2 + \sum_{i=1}^{n+m}\{\widehat{I}_{i,0}^{\mathrm{DR2}}(t)\}^2.$$

A.4. Addition simulation studies

A.4.1. Simulations when the true sampling score model, censoring model and failure outcome model each includes a different subset of covariates in X

To confirm that the simulation results and patterns are similar when a different subset of covariates affected one model but not others, we carried out additional simulations when a different covariates are included in each of the true sampling score model, true censoring model and the true failure outcome model. Specifically, based on the covariates $\mathbf{X}$ generated in Section 5.1, we assume the true sampling score model only includes X_2 and X_3, that is,

$$P(S_i = 1|\mathbf{X}_i) = \frac{\exp(-6.7 - 0.4X_{i2} - X_{i3})}{1 + \exp(-6.7 - 0.4X_{i2} - X_{i3})}.$$

Further we assume the true censoring model only includes X_1 and X_3, that is,

$$\lambda_1^C(t|\mathbf{X}_i, S_i = 1) = \lambda_0^C(t|\mathbf{X}_i, S_i = 1) = \exp(0.1X_{i1} - 0.6X_{i3}).$$

All components of $\mathbf{X}$ are included in the true failure outcome model, and the remaining parameter specifications are identical to those under the strong sampling, covariate-dependent censoring scenario in Table 2 of the main manuscript.

When evaluating the performances of IPW and DR estimators, the correct sampling score model is fitted using covariates (X_{i2}, X_{i3}) whereas the incorrect model is fitted using X_{i3} as an illustration. For the failure outcome model (the model for $T_i|\{S_i = 1, \mathbf{X}_i\}$), the correct outcome model is fitted by Cox regression with (X_{i1}, X_{i2}, X_{i3}), whereas the incorrect outcome model is fitted using the log-normal accelerated failure time (AFT) model with (X_{i1}, X_{i2}, X_{i3}). For the censoring model, the correct model is fitted using Cox regression with (X_{i1}, X_{i3}) whereas the incorrect model is fitted

using only X_{i3}. Estimation results from WKM, IPW and DR estimators under strong sampling, covariate-dependent censoring scenario are provided in the following Web Table 1. It can be seen that the results from each estimators are similar to those from Table 2 of main manuscript and still demonstrate that DR estimators can improve upon IPW estimators.

A.4.2. Addition simulations when survey weights are ignored

To illustrate the necessity of accounting for the complex survey weights in the population data for IPW and DR estimators, we have carried out an additional set of simulations when survey weights are ignored in each estimator we compared in the original simulation. Briefly, we repeat the simulations in Table 2 of the main manuscript under strong sampling and covariate-dependent censoring but now ignore the survey weights in each estimator. In other words, we set $p_i = 1$ for each observation in the combined sample to illustrate the consequence of not adhering to recommendations in Table 1 of the main manuscript. The results in the following Web Table 2 show that all estimators exhibit substantial bias and the DR estimators can be even more biased than the IPW estimators. This is not unexpected because DR estimators make heavier use of the survey weights than IPW estimators, and ignoring the survey weights can lead to a greater distortion of its point estimate. This observation highlights the need to incorporate survey weights when the covariates in the target population are observed only through a non-random sample of all non-participants.

References

Bai, X., Tsiatis, A. A., and O'Brien, S. M. (2013). Doubly-robust estimators of treatment-specific survival distributions in observational studies with stratified sampling. *Biometrics*, 69(4):830–839.

Chen, P.-Y. and Tsiatis, A. A. (2001). Causal inference on the difference of the restricted mean lifetime between two groups. *Biometrics*, 57(4):1030–1038.

White, H. (1982). Maximul Likelihood Estimation of misspecified models. *Econometrica*, 50(1):1–25.

A.5. Web Tables and Figures

Web Table 1: Additional simulation results for estimating TASTE at three specific target survival times under the strong sampling, covariate-dependent censoring scenario, when each of the true sampling score model, censoring model and failure outcome model contains a different set of covariates. The columns for T, S and C indicate whether the model for T, S, or C, is correctly (T) or incorrectly (F) specified. All values under Bias, ESD, ASE and CP columns are multiplied by 100.

Method	T	S	C	Bias	ESD	ASE	CP	Bias	ESD	ASE	CP	Bias	ESD	ASE	CP
				$t = 0.128$ and $\delta(t) = -0.077$				$t = 0.331$ and $\delta(t) = -0.141$				$t = 0.712$ and $\delta(t) = -0.178$			
WKM		T		0.42	2.6	3.0	96.7	0.17	3.6	5.0	99.3	-0.86	4.3	8.1	100.0
		F		0.37	2.6	3.0	96.8	0.09	3.6	5.0	99.4	-0.96	4.2	8.0	99.9
IPW1		T	T	3.87	8.9	12.2	97.9	3.87	8.4	11.7	97.8	3.64	7.9	11.0	97.5
		F	T	3.86	8.9	12.2	97.9	3.86	8.4	11.7	97.8	3.58	7.9	11.0	97.6
		T	F	4.00	8.7	12.1	97.8	4.00	8.2	11.6	97.6	3.78	7.7	10.9	97.3
		F	F	4.00	8.7	12.0	97.9	4.00	8.2	11.6	97.7	3.82	7.7	10.9	97.3
IPW2		T	T	3.88	6.4	10.5	98.4	3.88	6.8	10.7	98.2	3.66	7.1	10.5	97.8
		F	T	3.87	6.4	10.5	98.4	3.87	6.8	10.7	98.2	3.59	7.1	10.4	97.8
		T	F	4.01	6.2	10.4	98.3	4.01	6.6	10.5	98.2	3.80	6.9	10.3	97.6
		F	F	4.00	6.2	10.4	98.3	4.00	6.6	10.5	98.3	3.83	6.9	10.3	97.6
DR1	T	T	T	0.04	2.8	2.6	93.2	0.00	3.7	3.5	94.0	0.01	4.1	4.0	94.4
	T	F	F	0.04	2.8	2.6	93.1	0.00	3.7	3.5	94.0	0.02	4.1	4.0	94.4
	F	T	T	0.04	3.0	3.0	95.2	-0.01	3.9	3.9	95.3	-0.03	4.3	4.3	95.1
	F	F	F	0.04	2.8	2.6	93.4	0.22	3.7	3.5	94.0	0.19	4.1	4.0	94.4
DR2	T	T	T	0.04	2.8	2.6	93.0	0.00	3.7	3.4	93.5	0.01	4.1	3.9	93.7
	T	F	F	0.04	2.8	2.6	92.9	0.00	3.7	3.4	93.5	0.02	4.1	3.9	93.8
	F	T	T	0.04	3.0	3.0	94.9	-0.01	3.9	3.8	94.9	-0.02	4.3	4.2	94.5
	F	F	F	0.04	2.8	2.6	93.0	0.22	3.6	3.4	93.2	0.19	4.1	3.9	93.8

Web Table 2: Additional simulation results for estimating TASTE at three specific target survival times under strong sampling, covariate-dependent censoring scenario when the survey weights are ignored in each estimator. The columns for T, S and C indicate whether the model for T, S, or C, is correctly (T) or incorrectly (F) specified. All values under Bias, ESD, ASE and CP columns are multiplied by 100.

Method	T	S	C	Bias	ESD	ASE	CP	Bias	ESD	ASE	CP	Bias	ESD	ASE	CP
				$t = 0.128$ and $\delta(t) = -0.077$				$t = 0.331$ and $\delta(t) = -0.141$				$t = 0.712$ and $\delta(t) = -0.178$			
WKM		T		4.58	6.1	6.0	86.9	7.21	8.0	9.6	90.3	6.64	8.7	14.9	98.3
		F		-3.52	4.1	4.6	94.1	-6.95	5.1	7.6	95.9	-10.22	5.5	11.9	99.4
IPW1		T	T	-1.39	31.4	24.8	98.1	-1.39	30.3	23.0	98.4	6.00	29.0	20.1	97.0
		F	T	-4.42	20.6	17.4	98.8	-4.42	20.2	16.5	98.2	-7.19	19.4	15.1	98.0
		T	F	4.20	22.2	20.6	97.2	4.20	20.7	18.8	96.5	6.82	18.9	16.3	95.6
		F	F	-1.90	16.2	16.8	98.7	-1.90	15.5	15.9	99.0	-8.95	14.7	14.5	98.3
IPW2		T	T	-1.51	26.8	21.1	97.4	-1.51	27.0	20.9	97.9	5.75	26.6	19.1	97.1
		F	T	-4.39	18.6	15.2	99.0	-4.39	19.0	15.2	98.0	-7.18	18.9	14.5	97.0
		T	F	4.11	18.2	17.2	96.8	4.11	18.3	17.0	95.7	6.62	17.6	15.5	95.4
		F	F	-1.87	13.6	14.5	99.1	-1.87	14.0	14.5	99.1	-8.94	14.0	13.9	97.4
DR1	T	T	T	5.26	6.6	5.9	81.7	9.76	8.8	7.9	69.6	12.44	9.8	8.9	62.1
	T	F	F	5.31	4.1	3.9	69.5	9.73	5.5	5.0	49.3	12.27	6.2	5.4	38.6
	F	T	T	5.25	6.6	6.0	81.6	9.78	8.9	8.1	71.1	12.50	10.0	9.1	65.2
	F	F	F	7.05	4.3	3.9	54.2	9.10	5.6	5.0	53.5	7.98	6.1	5.4	65.8
DR2	T	T	T	5.26	6.2	5.5	79.9	9.76	8.3	7.4	67.0	12.41	9.3	8.3	60.9
	T	F	F	5.32	4.1	3.8	68.8	9.73	5.4	4.9	47.9	12.27	6.2	5.2	37.1
	F	T	T	5.27	6.2	5.5	79.8	9.77	8.3	7.4	68.6	12.43	9.5	8.5	63.2
	F	F	F	7.07	4.3	3.8	53.0	9.10	5.6	4.9	51.9	7.98	6.1	5.3	64.5

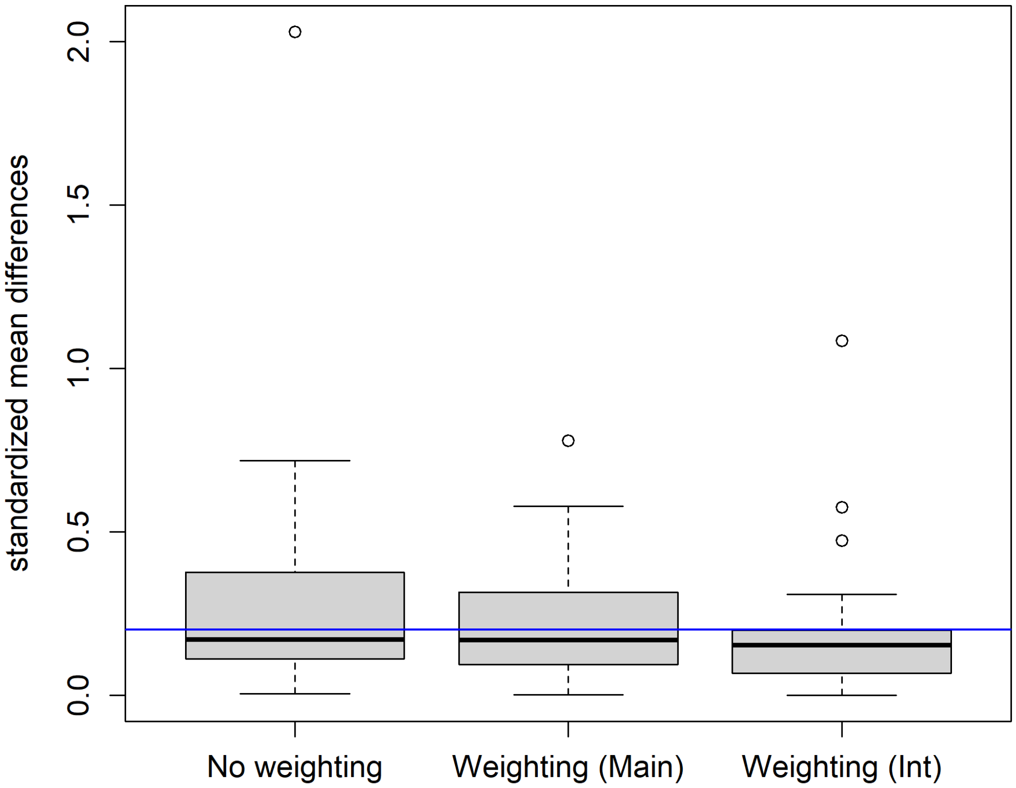

Web Figure 1: Standardized mean differences (measuring covariate balance between the RCT data and survey weighted observational data) for each covariate before and after inverse odds of sampling weighting. Main: the sample score model is estimated by logistic regression with main effects. Int: the sample score model is estimated by logistic regression with main effects, interactions between race/ethnicity and BMI, as well as an interaction between years of diabetes and SBP.
